# Ice Nucleation and Freezing Consequences in Perennial Plants

**DOI:** 10.1111/ppl.70961

**Published:** 2026-06-07

**Authors:** Lia Lamacque, Nicolas Dusart, Pierre Amato, Katline Charra‐Vaskou, Stephen Ingram, Anna Lintunen, Cindy E. Morris, Gilbert Neuner, Matthias Stegner, Guillaume Charrier

**Affiliations:** ^1^ INRAE Plant Pathology Montfavet France; ^2^ INRAE, UR P3F Lusignan France; ^3^ Université Clermont Auvergne, INRAE, PIAF Clermont‐Ferrand France; ^4^ Laboratoire Microorganismes : Génome et Environnement, LMGE Clermont‐Ferrand France; ^5^ Institute for Atmospheric and Earth System Research/Physics University of Helsinki Helsinki Finland; ^6^ Institute for Atmospheric and Earth System Research/Forest Sciences University of Helsinki Helsinki Finland; ^7^ Department of Botany University of Innsbruck Innsbruck Austria

**Keywords:** freezing damage, ice nuclei, ice propagation, plant tissues, winter embolism

## Abstract

The precise location where ice forms in plants affects the physical constraint it exerts on the different biological compartments (cells, tissues, organs). It is therefore critical to understand where and how ice nucleates to predict the extent of low temperature damage. On one hand, extracellular ice formation can protect living plant cells by lowering their intracellular freezing point through water efflux and an increase in osmolyte concentration. On the other hand, extended freezing‐induced dehydration may cause damage and rupture of the plasma membrane. The location and pattern of ice formation in plants are marked by high spatio‐temporal variability in relation to the type of plant tissue, its developmental stage, and the nature of the initial ice nucleus. This review focuses on the mechanisms and dynamics of intrinsic ice nucleation and subsequent propagation in perennial plants. We describe the factors that influence ice nucleation, such as the nature of nucleating agents and other biophysical conditions. We also highlight the shortcomings of studies on plant freezing, especially regarding laboratory studies, and emphasize the need to investigate ice nucleation in plants using interdisciplinary approaches. We finally provide a practical workflow to guide new experimenters in this research field.

## 1 Introduction

**FIGURE 1:**
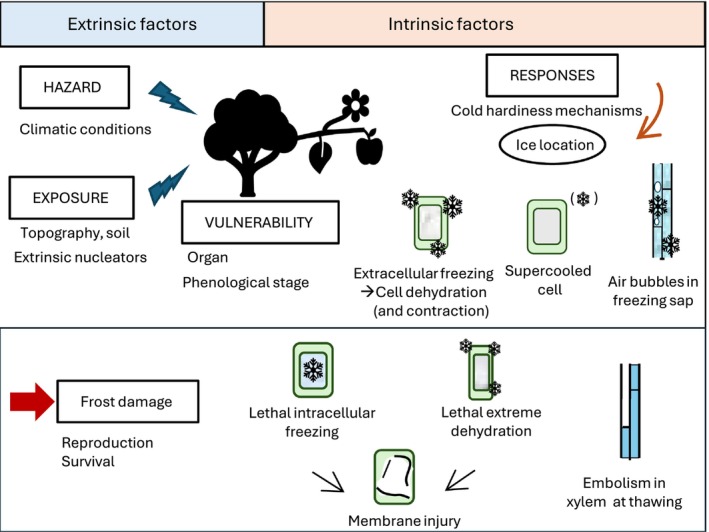
Overview of freezing risk for plants. Freezing risk is determined by the interaction of four components: hazard, exposure, vulnerability and response (Simpson et al. 2022). Extrinsic conditions (climate, topography, nucleators) and intrinsic traits (organ type, phenological stage, cold hardiness mechanisms) modulate the plant's response to freezing. The location of ice formation (represented by snowflakes) plays a crucial role in cold hardiness mechanisms: in ice‐tolerant tissues, extracellular freezing leads to cell dehydration, or supercooling, where ice can still form outside the cells without causing freeze‐induced dehydration. Freezing damage arises when intracellular ice forms, when extreme dehydration cannot be tolerated, and impeded sap flow by embolisms triggered during the thawing of frozen xylem sap, ultimately impairing plant survival and successful reproduction.

Low, and notably subzero, temperatures are a limiting factor for plant development and survival, and this is therefore related to the altitudinal and latitudinal species distribution, and agricultural yields (Parker 1963; George et al. 1974; Ashworth 1992; Neuner et al. 2020). Ice formation induces freezing stress by inducing cellular and hydraulic damage (Charrier et al. 2013; Figure 1). However, frost events (i.e., the occurrence of negative air temperature) do not necessarily imply freezing (i.e., the change of physical state of water). Climatic conditions (e.g., wind, humidity and temperature) define the frost hazard (Figure 1) by determining the type of frost: advective or radiative (Box 1, Snyder and Melo‐Abreu 2005). Topography, soil characteristics and plant cover define the frost exposure by setting local temperature gradients and organ temperature (Snyder and Melo‐Abreu 2005). Freezing in plants is also controlled by the presence of ice nuclei, because ice is typically initiated around a particle and then propagated in plant tissues (Vali 1995). However, ice nuclei are usually under‐considered in frost risk studies, where damage is often simply related to frost exposure and meteorological hazard.

BOX 1 **Concepts of frost in meteorology.**

The different types of frost from a meteorological perspective are described (Snyder and Melo‐Abreu 2005):

Radiative frost occurs on clear nights (no clouds), with no wind, and when a vertical temperature inversion is present (temperatures rise with altitude). These conditions allow for greater radiative heat loss (energy loss) from the ground and plant, with minimal loss by convection and transpiration. The vertical temperature inversion generally occurs between 9 and 60 m (Perry 1994) and can therefore affect tall trees.

On the contrary, advective frost occurs when conditions are very windy, with cold air masses. These wind conditions do not allow for temperature inversions because the atmosphere is well mixed. Temperature can drop below 0°C during the day. These windy conditions also increase plants' evapotranspiration, exacerbating water loss and speeding organ cooling.

Schematic impact of radiative and advective frost on plant energy budget major fluxes, Net radiation (Rn) and sensible heat (H) also referred to as convection.
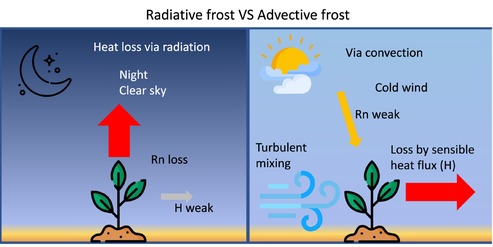

The temperature and rate at which freezing occurs influence the stress caused by freezing and thus the temperature at which lethal damage happens (Olien 1971). The negative effects of ice nucleation can be of two types: (1) cellular damage, when dehydration and rehydration are too extreme or too fast and/or when intracellular freezing occurs (Figure 1, Stuckey and Curtis 1938; Burke et al. 1976); (2) hydraulic disruption, when gas embolisms occur due to the formation of air bubbles in ice and their expansion at thawing (Utsumi et al. 1998; Cruiziat et al. 2002; Charrier et al. 2014). In intracellular freezing, ice crystals destroy the membranes and the cells are irreversibly damaged (Mazur 1969; Burke et al. 1976). Intracellular ice formation is always lethal (Siminovitch and Scarth 1938; Levitt 1980). On a macroscopic scale, lesions lead to tissue softening, dieback and malformations (Sakai and Larcher 1987), reduce reproductive success (Rodrigo 2000; Ladinig et al. 2013), and impact biomass production (Bokhorst et al. 2008).

Freezing‐avoidance and freezing‐tolerance are two strategies used by plants to resist frost events (Levitt 1980). Seasonal avoidance allows many species to avoid harsh winters, for example, seed or belowground organs protected by the soil for annual herbaceous species. Another avoidance strategy is achieved by allowing water to remain in a metastable liquid state at negative temperatures, that is, supercooled (Levitt 1980; Wisniewski et al. 2003). Freezing tolerance is achieved in plants that acclimate to cold, for which ice formation in the apoplast has a protective effect. This review focuses on perennial plants, where ice formation within tissues is typically tolerated. In this case, apoplastic ice pulls water from the cells, due to the difference in water potential, where it freezes (Sakai and Larcher 1987). Although freezing releases heat, water efflux increases intracellular solute concentration and, if too intense, this causes osmotic shock to plant organelles and macromolecules that are bound or solvated by water molecules (Olien 1973). Cells thus resist freeze‐induced dehydration by maintaining fluidity of their plasma membrane (Yoshida 1986; Zhang et al. 2016) and increasing their solute content to protect molecular and cellular structures. The membrane composition (aquaporins, unsaturated fatty acids) ensures sufficient fluidity for water to exit during freezing and re‐enter during thawing (Uemura et al. 2006). Furthermore, the plasma membrane and cell wall prevent ice from penetrating the cell, and the adhesion between ice and the hydrophilic molecules that make up these structures (phospholipids, glycoprotein, cell wall hemicellulose and pectins) help to regulate the formation and propagation of ice (Chambers and Hale 1932; Olien and Smith 1977; Uemura et al. 2006). The high solute content allows cells to resist dehydration and their structure to remain fluid during freezing.

Throughout the year, although plants are exposed to seasonal changes in their physiology, how it affects ice nucleation activity (INA) is not fully understood, preventing an accurate prediction of freezing risk. Even a seasonal change in freezing resistance mechanisms can occur: freezing‐avoidance during periods of low frost exposure and freezing‐tolerance during colder periods (Venn et al. 2013; Sklenář 2017). However, the changing climate, especially the alteration of winter conditions with increasing late frost risk in temperate regions, is affecting the seasonality of plant freezing behavior and opening many new research avenues. This review aims to highlight the complexity of ice nucleation from the viewpoint of multiple disciplines to take advantage of existing knowledge in each field. An overview of ice nucleation and its difficult predictability could benefit future studies on freezing patterns and cold hardiness (CH). We also suggest forward‐looking insights and a methodology for conducting future studies.

## 2 Physics and Chemistry of Ice Formation

### 2.1 Fundamental Mechanisms of Ice Nucleation

Ice formation results from the organization of water molecules into an initial “critical” ice nucleus. If thermodynamic conditions are favorable, ice grows irreversibly, ensuring its subsequent propagation into a larger crystalline structure. The organization of water molecules to form a precritical ice nucleus can occur either via random fluctuations in density and orientation of water molecules in the bulk liquid, or via the help of a catalyzing surface. To ensure the subsequent propagation of a massive crystalline structure, the initial ice crystal faces energetic barriers, described below, whose magnitude decreases mainly with temperature (Olien 1974). Specifically, this growth is ruled by the energy balance between attachment and detachment of water molecules between initial ice clusters and liquid phase relative to the overall energy budget of the bulk system. In pure water, that is, without any solutes, an initial ice crystal of critical size to permit ice growth at near −39°C requires the clustering of roughly 70 water molecules whereas it requires 45,000 molecules at −5°C (Vali 1995). This phenomenon brings to light supercooling, which is the process by which water remains in a liquid state at sub‐zero temperature. Hence, under most environmental conditions in which perennial plants live, random organization of water molecules into an effective initial ice crystal is extremely improbable. In these plant tissues, the triggering of ice formation during frost events is therefore the result of the action of heterogeneous ice nuclei: solid materials that are in contact with the water in or on plant tissues as it supercools to temperatures where the ice nuclei decrease the energy barriers to crystal propagation (Hoose and Möhler 2012; Vali et al. 2015). In theory, a single nucleus is sufficient to freeze a contiguous mass of water and this is a prerequisite for freezing an entire tissue at negative temperature. However, several independent nucleation events can also occur simultaneously. Spatial heterogeneity of INA is a common phenomenon in plants, particularly given that environmental (e.g., temperature gradients, soil and weather conditions) and physiological factors (e.g., phenology and avoidance mechanisms) vary spatially and temporally.

The temperature at which heterogeneous ice nuclei can effectively catalyze ice formation depends on the size of the specific nucleation site on the molecular surface and the surface properties such as shape and hydrophobicity (Burke and Lindow 1990; Fitzner et al. 2015; Bi et al. 2017). Numerous inert and biological materials, which are notably present in the atmosphere, have the capacity to nucleate ice (Murray et al. 2012) in a temperature range from a few degrees below 0°C to near the spontaneous ice nucleation temperature of pure water (approx. −39°C). The most studied materials include feldspar (mineral dust; Atkinson et al. 2013), cellulose (Hiranuma et al. 2015), pollen (Diehl et al. 2001; Pummer et al. 2012; Hader et al. 2014), and microorganisms such as diatoms (Knopf et al. 2011), fungi (Pouleur et al. 1992; Fröhlich‐Nowoisky et al. 2015), and bacteria, notably *Pseudomonas syringae* (Maki et al. 1974; Upper and Vali 1995; Cochet and Widehem 2000; Morris et al. 2000; Joly et al. 2013; Lindow 2023). Lignin has an INA of −18°C (Bogler and Borduas‐Dedekind 2020) and cellulose about −21°C (Hiranuma et al. 2015), while *Pseudomonas syringae* is a more active agent, with an INA of −2°C (Govindarajan and Lindow 1988). Given the extensive literature on ice nucleation active bacteria, this review focuses instead on the intrinsic controls of ice formation in plants.

Ice nuclei help to overcome the activation barrier to the transition from liquid water to ice in heterogeneous freezing (Kashchiev 2000). The source of this barrier is the interfacial free energy between ice (the more thermodynamically stable phase) and supercooled water (the metastable phase). For heterogeneous ice nuclei, it has not been possible to measure the interfacial energy (Maeda 2021), but based on theory, the effectiveness of heterogeneous ice nuclei lies in how they orient the hydrogen bonds (dipoles) of water molecules. Substrates that are poor nucleators orient dipoles in parallel with each other thereby reducing entropy and raising the free energy of any potential nuclei growing on the substrate (Fletcher 1959). This mechanism has displaced “matching lattices” as the underlying framework to understand heterogeneous ice nucleation (Maeda 2021). Nevertheless, despite a strong theoretical thermodynamic framework for the phase change from liquid water to ice, the ice nucleation effectiveness of various substances is difficult to explain (Maeda 2021). One reason for this is that the nanoscale topography and surface roughness can be influential in nucleation efficiency, thereby adding uncertainty to predictions. Heterogeneous ice nucleation is not only dependent on the ambient temperature and ice nuclei but also on the concentration and nature of solutes in the tissue. For a given solute in water, the equilibrium freezing point (i.e., the melting point) decreases with increasing concentration. The influence of solutes on the ice nucleation temperature of pure water can be generalized to values independent of their nature by considering their impact on water activity (osmolality; Zobrist et al. 2008). Most solutes cause a depression in the equilibrium freezing point, but their influence is not simply based on their osmotic potential, as they can activate or deactivate ice nucleation sites depending on their nature. Different chemical treatments can modify the INA in various ways. For example, acidic pH or gas exposure (NO_2_, O_3_) decreases the INA of certain bacterial strains (Attard et al. 2012), while dissolved ammonium sulfate increases the INA of quartz or kaolinite (Whale et al. 2018; Worthy et al. 2021). This suggests that different ice nucleation mechanisms occur on different ice nuclei (Whale et al. 2018).

When ice crystals expand from the initial nucleus, molecules and ions other than water tend to be excluded from crystals and progressively concentrate in the surrounding liquid phase (Degrandpre et al. 2021; Wang et al. 2016). This phenomenon creates a strong osmotic gradient between the liquid and frozen phases, associated with water and solute fluxes, which also occur in the opposite directions during thawing. These fluxes can be highly damaging to cells by rapid modifications of cell turgor. For this reason, aquaporins, structures dedicated to transport water through membranes, likely play a major role in cryoprotection in different organisms (Philip et al. 2011; Tong et al. 2019; Rahman et al. 2020).

### 2.2 Ice Nucleation Activity of Intrinsic Plants Ice Nuclei

Ice nuclei can be found within or on the surface of plant tissues; therefore, leading to internal or external ice nucleation (Box 2). An intrinsic nucleus is part of the plant itself or a physical particle produced by the plant. It is important to distinguish between ice nuclei, as a particle responsible for ice nucleation, and INA, which describes the process of heterogeneous ice nucleation in a tissue and is given in temperature units (Ishikawa 2014). INA can thus be measured whatever the origin of the ice nucleus.

BOX 2 **Vocabulary difference between external, internal, extrinsic and intrinsic nucleation.**

*External Ice Nucleation*:

Ice nucleation is initiated outside the plant, typically on the surface of an organ such as a leaf, stem, or bud. This process often involves extrinsic nuclei, such as ice‐nucleating bacteria, dust particles, as well as water in the form of dew droplets because these can be deposited on the surface of plants. However, intrinsic nuclei can also theoretically be involved in external nucleation.

*Internal Ice Nucleation*:

Ice nucleation occurs within the plant tissues, either extracellularly (e.g. in the apoplast) or intracellularly (inside the cell itself). It can be initiated both by intrinsic and extrinsic nuclei, for example, if bacteria have entered the tissue.

A misuse of terminology can lead to confusion between terms, as external and extrinsic, or internal and intrinsic, are often used as synonyms. However, ice nucleation can be internal and extrinsic when *Pseudomonas syringae* nucleate freezing inside the plant (Roos and Hattingh 1983). External intrinsic nucleation can be observed on leaf trichomes (Gorb and Gorb 2022).

Intrinsic plant ice nuclei (Wisniewski et al. 2009; Ishikawa 2014; Ishikawa et al. 2018) have been less extensively described in literature than extrinsic ice nuclei like the INA bacteria *Pseudomonas syringae* for which the gene coding for the ice‐nucleating protein and its structure are well known (Maki et al. 1974; Pandey et al. 2016; Lindow 2023). Producing intrinsic ice nuclei is directly related to the strategy used by the plants: high INA implies that freezing‐tolerance is more likely at play, whereas low INA (freezing point at colder temperatures) may rather be a freezing‐avoidance strategy by favoring supercooling. The seasonal dynamics of INA, through the abundance, nature, and location of the intrinsic ice nuclei, thus define the freezing resistance mechanism at play (Ishikawa 2014; Kishimoto, Sekozawa, et al. 2014; Ishikawa et al. 2015, 2018). Indeed, INA can be considered to confer a protective role in ice formation in the meristematic tissues at the beginning of winter (Ishikawa 2014; Ishikawa et al. 2015, 2018). As we will see in the next section (barriers), the location of apoplastic ice can be important in protecting certain tissues, and therefore accumulating ice nuclei in these areas could be beneficial for the plant.

The chemical nature and precise locations in plant structures of intrinsic nuclei remains difficult to elucidate (Pearce 2001) and only a few have been identified: a protein in rye (Brush et al. 1994), a polysaccharide in succulent plants and pollen (Krog et al. 1979; Goldstein and Nobel 1991; Kinney et al. 2024). Other macromolecules, such as starch granules (polysaccharides) from herbaceous species (Bose et al. 2024) and lignin (Bogler and Borduas‐Dedekind 2020), were proposed to participate in ice formation in clouds. For instance, lignin has an INA around −18°C, which is well below the INA of leaf fragments (Steinke et al. 2020). So, what about these molecules in their intrinsic form within the plant? More broadly, what is the nature of intrinsic ice nuclei for plants? Ice nuclei are likely to be diverse, complex and variable according to species, varying throughout the year or over the plant lifespan. The thermolability of INA differs across tissues, suggesting that distinct types of ice nuclei are involved (Ishikawa et al. 2018). Furthermore, the INA of a compound may be solely due to its structure rather than offering any biological benefits, as has been suggested to be the case with the hydrophilicity of pollen grains (Kinney et al. 2024). Ice nuclei are affected by pre‐freezing conditions, for example, the exposure of branches to low temperature decreases their INA (Kishimoto, Sekozawa, et al. 2014). In addition, the very fine tridimensional structure of tissues, at the nanoscale, can also be a determining factor, simply by aspects of geometric shape that catalyze ice formation (Conrad et al. 2005; Maeda 2021).

Experimentally, INA in plants is measured by immersion freezing. Tissues are immersed in water and the temperature is gradually lowered until freezing occurs (Kishimoto, Sekozawa, et al. 2014; Kishimoto, Yamazaki, et al. 2014; Ishikawa et al. 2015, 2016, 2018). However, these tests cannot discriminate between extrinsic and intrinsic nucleation, as they only measure the bulk ice nucleation of the sample. The presence of intrinsic nuclei in plant tissues has been demonstrated through antibiotic treatments or autoclaving, with very high “residual” INA levels, around −1°C and −2°C (Gross et al. 1983, 1984, 1988; Kishimoto, Yamazaki, et al. 2014). However, the results of these treatments remain ambiguous, since bacterial cell fragments or proteins may remain active, as in the case of Snomax (Snomax LLC; Ward and DeMott 1989). In some cases, such as in wheat, it has been shown that microflora can influence the freezing sequence, demonstrating the complexity of nucleation (Livingston et al. 2021), perhaps combining extrinsic and intrinsic nuclei.

Future research should focus on developing methods for the isolation and identification of intrinsic ice nuclei. This major challenge lies in disentangling the uncommon ice‐nucleating properties of common compounds (such as lignin and cellulose) and exploring the less abundant ones whose functions remain unknown. Immersion freezing tests involving different treatments would allow the identification of the organic or inorganic nature of molecules involved. Organic molecules can be identified through heat treatment or hydrogen peroxide digestion, and protein, lipid, and other biological molecules can be further identified through targeted chemical or enzymatic treatments. Cycles of freezing and subdivision tests on droplets containing plant material can enable the isolation and subsequent analysis of ice nuclei, particularly with regard to their chemical composition. This approach allowed the identification and visualization of ice nuclei in soil particles (Hill et al. 2016). Crystallography, polarized light or confocal microscopy could also bring new insights into the process of ice nucleation (Deville 2017; Saint‐Michel et al. 2017).

## 3 Protecting Susceptible Tissues: The Role of Ice Barriers

Beyond the mechanisms governing ice nucleation itself, limiting the propagation of ice within the plant is crucial for its survival. The propagation of ice after the formation of the initial crystal can be very rapid, up to 24 cm s^−1^, depending on the propagation direction, the supercooling temperature, the tissues and the plant species (Single and Marcellos 1981; Zámećník et al. 1994; Wisniewski et al. 1997; Pearce and Fuller 2001; Hacker and Neuner 2008). The formation and propagation of ice can be prevented by certain molecules such as anti‐freeze proteins (AFPs; Wisniewski et al. 2020), or by the presence of barriers and/or the ability to promote ice formation in defined areas (Table 1). Furthermore, the physiological status of plant tissues, linked to water status and growth potential, influences the type of response and the extent of damage.

**TABLE 1:** Summary of barriers type at different plant scales.

Barriers type	Scale	Mechanisms
Thermal	Plant, organ	Soil coupling Insulation by snow cover Microclimatic thermal gradients within a single plant
Physical	Plant, organ, tissue, cellular	Complex bud scale architecture plant cuticle impermeability Internal anatomical barriers in stems/buds Temporary hydraulic disconnection Specialized xylem barriers Extra‐organ ice formation Plasma membrane and cell wall
Chemical	Cellular, tissue	Anti‐Freeze Proteins (AFPs) Formation of de‐methyl‐esterified homogalacturonans by calcium ions Lipophilic substances (e.g., triterpenoids and flavonoid aglycones)

Thermal gradients within the plant prevent ice propagation. Such thermal barriers are observed in high alpine cushion plants that remain unfrozen during summer night frosts due to thermal conduction from the warm soil and the thermal inertia of the stored water (Hacker et al. 2011). Although single flowers may freeze, ice cannot spread into other flowers through the warmer basal part of the plant (Hacker et al. 2011). In spring geophytes, warm soil protects the underground organs from freezing by limiting the penetration of ice from frozen above‐ground organs (Bertel et al. 2021). This type was also observed in alpine plants that generally have their meristems buried in the soil (Stegner, Lackner, et al. 2020; Körner 2021). Snow cover also protects small stature plants from fluctuating and extreme temperatures (Wipf and Rixen 2010).

Surface barriers also protect against extrinsic ice nucleation. Complex bud scale architecture in *Picea abies* (Kuprian et al. 2017, 2018) and impregnation with lipophilic substances (triterpenoids and flavonoid aglycones) in *Alnus alnobetula* (Neuner, Kreische, et al. 2019) prevent ice penetration from outside the bud. Hairy leaves and hairs within buds delay ice propagation by redirecting ice nucleation from the leaf surface to the trichomes, thereby maintaining an insulating boundary air layer (Gorb and Gorb 2022). The likelihood of extrinsic ice nucleation is low when leaf wettability is low; in fact, leaf wettability decreases with increasing elevation, where the frequency and intensity of frost increase (Aryal and Neuner 2010). The plant cuticle is generally considered to be impermeable to ice: droplets of a *Pseudomonas syringae* suspension freeze on the leaf surface without spreading inside (Wisniewski et al. 1997). Moreover, artificially applied hydrophobic particle films enhance the protection against extrinsic ice nucleation (Wisniewski et al. 2002).

Overwintering buds (Neuner, Monitzer, et al. 2019) or reproductive organs (Ladinig et al. 2013) are highly susceptible and can be immediately damaged upon contact with ice. Internal anatomical ice barriers act as physical barriers that prevent ice from entering these ice‐susceptible tissues from already frozen plant parts (Wisniewski et al. 2015). Such barriers are found in stems below vegetative buds of many temperate woody plant species (Neuner, Monitzer, et al. 2019), below reproductive bud tissues (Ishikawa and Sakai 1981, 1985; Ashworth and Davis 1984; Ashworth et al. 1992; Jones et al. 2000; Villouta et al. 2022) and in the flower stems of herbaceous (Bertel et al. 2021) and woody plants (Kuprian et al. 2014, 2016). Features of physical ice barrier tissues usually include provascular strands (no xylem connection), small, tightly packed cells with thick walls that lack vacuoles and intercellular spaces, regions of dry (desiccated) tissue, and mostly impregnation of cell walls with phenolic compounds (suberin and lignin) and accumulation of unesterified pectins (e.g., Kuprian et al. 2016). These chemical components reduce cell wall pore size and water in small cell wall pores does not freeze (Ashworth and Abeles 1984). In case of intact xylem connection, pit pore diameter has been shown to be reduced in the ice barrier zone (Kuprian et al. 2016). A temporary hydraulic disconnection in winter also acts as a barrier between stem and buds (Villouta et al. 2022). Therefore, the re‐establishment of xylem hydraulic continuity at growth resumption removes ice barriers (Ashworth 1984; Ashworth et al. 1992; Neuner and Beikircher 2010; Pramsohler and Neuner 2013). However, in the flower stem of *Calluna vulgaris* , despite an intact xylem connection, a specialized ice barrier within the xylem of the pedicel protects the ice‐susceptible flowers (Kuprian et al. 2016). Physical barriers are therefore key to restricting ice formation to specific areas where its accumulation can protect susceptible organs through “extra‐organ freezing” or “extra‐tissue freezing” (Sakai 1979; Ishikawa and Sakai 1982; Ashworth et al. 1988; Willick et al. 2019). Scale layers, hydrophobic interfaces, air gaps, or low‐conductivity tissues block ice penetration into ice‐susceptible bud tissues. Consequently, in winter buds, ice forms in spaces surrounding the bud; this can be in intercellular spaces of the subtending stem and/or bud scales or, as in *Alnus alnobetula* in the space between the young leaves, which are coated with lipophilic substances (Ishikawa and Sakai 1982; Endoh et al. 2014; Neuner, Kreische, et al. 2019). Large blocks of ice form under the epidermis or between the epidermis and the parenchyma in the petioles of freezing‐tolerant plants (McCully et al. 2004; Ishikawa et al. 2015, 2022; Stegner, Wagner, and Neuner 2020; Stegner, Lackner, et al. 2020). Extra‐organ ice is formed by pulling cellular water, which releases mechanical strain and prevents intracellular ice formation (McCully et al. 2004; Endoh et al. 2014). At cellular levels, the plasma membrane and the cell wall act as barriers against extracellular ice in freezing‐tolerant cells (Chambers and Hale 1932; Yamada et al. 2002). In cell walls, the formation of cross‐linkages between pectins (Hiraki et al. 2018) and de‐methyl‐esterified homogalacturonans by calcium ions is involved in ice protection (Villouta et al. 2022).

## 4 Tissue‐Specific Ice Nucleation and Seasonal Dynamics of Freezing Processes

### 4.1 Consequences of Ice Nucleation in the Xylem

In plants, water is transported from the root system to the leaves, stems, and buds through an apoplastic network, including the xylem. Low solute concentration and large water volume in the xylem increase the probability of ice nucleation (Asahina 1956). Ice also easily propagates in the xylem (Charrier et al. 2017). Despite these observations, predicting the temperature and the rate at which extracellular ice forms in vascular plants is complicated by the simultaneous presence of liquid, solid, and gaseous phases in the xylem and surrounding area during the freezing process. It is unclear how xylem sap remains hydraulically stable in the presence of dissolved gases above its freezing point, let alone below. Recent research suggests that the metastability of water under negative temperature or pressure has mixed effects on the freezing process: Freezing can cause bubbles to form (see below), and bubbles can initiate or suppress freezing (Ingram et al. 2025).

Extracellular freezing generates a substantial water driving force toward the ice front due to the water potential (Ψ) difference between the ice and liquid sap, leading to significant water fluxes within the stem (Palta et al. 1977; B. Cinotti 1990, 1991; Steponkus and Webb 1992; Charra‐Vaskou et al. 2016). The extremely low Ψ of ice at any specific freezing temperature can be determined using the Clausius–Clapeyron relationship (Rajashekar et al. 1982, 1983; Guy 1990):

(1) ΔΨT=–1.16T

where ΔΨ*T* (MPa) represents the difference in Ψ between two compartments at temperature *T*, and *T* (°C) denotes the temperature below the effective freezing point.

This equation illustrates that living cells experience significant dehydration as temperatures decrease (−1.16 MPa K^−1^). The low Ψ caused by ice can thus account for the considerable shrinkage observed in the stem during freezing events (B. Cinotti 1990; Zweifel and Häsler 2000; Améglio et al. 2001; Sevanto et al. 2006). The intracellular water in the bark cells and xylem parenchyma is drawn toward the ice front in extracellular lacunae and cell water will diffuse through the plasma membrane to the extracellular ice. Thus, the living cells will contract, creating a strong shrinkage of mainly the bark (approx. 70%) but also xylem tissue (approx. 30%) and therefore of the stem (Sakai and Larcher 1987; Zweifel and Häsler 2000; Améglio et al. 2001; Charra‐Vaskou et al. 2016, 2023; Lintunen et al. 2017).

Frozen xylem sap, when critical low temperature thresholds are surpassed, can damage living cells by cytorrhysis (freezing‐induced dehydration) or intracellular ice formation (Ristic and Ashworth 1993). Freezing and thawing can also cause embolism in xylem and, as a consequence, loss of xylem hydraulic conductivity (Sperry and Sullivan 1992; Mayr and Charra‐Vaskou 2007; Mayr et al. 2020). Embolisms form because gases dissolved in the xylem sap are not soluble in ice and thus form bubbles (Lintunen et al. 2022) that then expand and fill the conduits with air during thawing (Sperry and Sullivan 1992; Charra‐Vaskou et al. 2016, 2023). The fate of gas bubbles during thawing, that is, whether they dissolve or expand to embolize xylem conduits, is dependent on the water potential of the surrounding xylem sap during thawing (Mayr and Sperry 2010) as well as on the bubble size (Pittermann and Sperry 2006). Although the size of the bubbles is crucial in winter embolism formation, the critical factors affecting the bubble formation are unclear (Charrier et al. 2015; Lintunen et al. 2020, 2022; Charra‐Vaskou et al. 2023); the vulnerability to winter embolism increases with increasing conduit size (Davis et al. 1999; Pittermann and Sperry 2003; Charrier et al. 2014), but it has been suggested that also the water status of the tree (Mayr et al. 2006; Lintunen et al. 2022) and the amount of gases escaping from the stem (Lintunen et al. 2014, 2022) during the freezing process plays a role in the bubble formation. Repeated freeze–thaw cycles have also been shown to influence winter embolism vulnerability (Mayr et al. 2006).

### 4.2 Influence of Plant Water Status on the Freezing Processes Across Seasons

In summer, well‐hydrated, non‐acclimated trees typically have low solute concentration in their living cells and thus a less negative ice nucleation threshold that may increase their vulnerability to intracellular freezing (Sakai and Larcher 1987). Thus, a sudden temperature drop typically leads to significant cellular damage (Lintunen et al. 2015), resulting in cell mortality and potential plant death. In winter, acclimated trees have lower water content (Chen et al. 1976; Ögren 1999; Gusta et al. 2004) and higher solute concentration, which increase the osmolarity and thus decreases the intracellular temperature threshold for freezing (Towill and Mazur 1976; Levitt 1980; Améglio et al. 2006).

Although apoplastic freezing in the extracellular compartment is influenced by plant water status (Lintunen et al. 2018), the reasons for this are just beginning to be understood. Xylem water potential can be decomposed into contributions from both the physical negative pressure pulling the water upwards and the osmotic pressure caused by the presence of solutes (Sevanto et al. 2012). As mentioned above, osmotic contributions from inorganic salts suppress the melting point (Koop et al. 2000). This is the physical basis, for example, salting roads during the winter, causing snow to melt: plants appear to practice a version of this when promoting extracellular freezing over intracellular.

In theory, negative pressure should cause melting points to rise, although there is growing evidence that the reverse is true (Arias et al. 2017; Lintunen et al. 2018). It is therefore possible that water potential itself acts as a sort of antinucleator, through an unidentified mechanism. In contrast to living cells, the apoplastic compartment expands as its water freezes. This expansion may somewhat release the tension in the remaining liquid, as it is compressed back toward its equilibrium density by the ice front. Negative pressure is also capable of air seeding bubbles from embolized conduits into adjacent water‐filled conduits (Schenk et al. 2015; Jochen et al. 2017) and generating acoustic emissions (Charrier et al. 2014). These bubbles may act as seeds of further embolism, seeds of ice formation, or they simply transport gas further up the water column. The risk of embolism formation therefore increases when water potential is low prior to freezing (due to more and larger bubbles in the sap) and at thawing (bubbles are not likely to redissolve). In the absence of other intrinsic nucleators, the xylem conduit walls are likely to play a role in catalyzing freezing, and this process would be suppressed by the presence of bubbles (Ingram et al. 2025), making them also strong candidates for an antinucleator. However, bubbles formed by air seeding across pits, those nucleated at the ice front by changes in solubility, and those nucleated in cavities on the conduit surface may each be distinct classes of bubbles with different properties. It is likely that all bubble types are capable of expanding into emboli (Schenk et al. 2015; Lintunen et al. 2022; Ingram et al. 2024) but it is not known whether they can each act as antinucleators.

Several uncertainties still remain: if freezing is suppressed by bubbles, and bubble formation is initiated by freezing, how do these two processes interact with each other? Does water potential indirectly affect the concentrations of metabolites (e.g., flavonoids, triacylglycerides) or AFPs in xylem sap that could alter its freezing behavior? What are the nanoscale surface features of lignified xylem walls, and do they render it hydrophobic or hydrophilic at that scale? We recommend high‐precision techniques, like mass spectrometry and atomic force microscopy, be deployed to help answer these questions. It may also be necessary, in the coming years, to extend or amend the cohesion tension theory so as to more directly account for the presence of additional phases of matter in the xylem system.

### 4.3 From the Stem to the Bud: Seasonal Transitions and Tissue‐Specific Ice Nucleation

Understanding ice formation and propagation in perennial plants requires an integrated spatio‐temporal perspective that accounts for differences between tissues over the season. Meristematic tissues (buds, cambium) and reproductive structures (flowers, fruits) exhibit distinct patterns of vulnerability due to changes in INA and CH (Box 3). These changes correspond to physiological transitions that occur during specific phases: from the growing season to the dormant period (bud set, growth cessation and endodormancy induction; Charrier et al. 2023), and from the dormant period to the growing season (ecodormancy release). These seasonal changes are closely linked with water status, sugar content, and the establishment of ice barriers. Although more attention has been given to the dynamics of freezing tolerance than to avoidance, freezing vulnerability can also be related to the loss of the capacity to prevent ice nucleation (Wisniewski et al. 2009) and propagation (Villouta et al. 2020, 2022).

At the branch scale, vascular tissues such as xylem and phloem show distinct responses due to their anatomical structure and physiological function. Many studies investigate freezing tolerance by separating bark (including phloem and cambium) and xylem, as these tissues are more accessible. For example, xylem tissue deacclimates more rapidly and presents higher damage than bark cells for the same ice nucleation temperature (−3°C) in *Betula platyphylla* (Takeuchi and Kasuga 2018). In *Vitis vinifera* , the xylem was more cold hardy than the phloem as the deacclimation of the phloem was associated with a decrease in soluble carbohydrates and an increase in water content (Gonzalez Antivilo et al. 2020). Ice nucleation in woody stems initiates in the youngest xylem or the cambium–phloem region, and ice rapidly spreads to the rest of the stem (Neuner et al. 2010; Charra‐Vaskou et al. 2016).

BOX 3 **Cold hardiness and ice nucleation: decoding the freezing response in plants.**

Cold hardiness mechanisms are defined in five categories according to Wisniewski et al. (2003): timing of cold acclimation onset, the rate of acclimation, the maximum level of freezing tolerance achieved, its maintenance during winter, and the rate of deacclimation in spring. Among these, cold hardiness (CH) is the temperature that the cell can resist and it is also a dynamic trait that varies seasonally in response to changing environmental conditions. The temperature of CH is commonly quantified using the lethal temperature for 50% of samples (LT50).

Cold hardiness can differ from ice nucleation temperature (INT). The solidification of water is exothermic, releasing latent heat (McLeester et al. 1969; Olien 1973). The detection of latent heat indicates the freezing of plant tissues (INT). Exotherms are detected using thermocouples, infrared cameras, or thermoelectric modules. Two key exothermic events can be detected in plants: the high temperature exotherm (HTE) and the low temperature exotherm (LTE), corresponding to extracellular and intracellular ice nucleation, respectively (Brown and Reuter 1974, Pramsohler et al. 2012). The lethal temperature for 50% of samples (LT50) has been shown to correlate with the LTE in many species/organs in the case of supercooling (Kaku and Iwaya 1979). In freezing‐avoidant or unacclimated plants, a single exothermic peak is usually observed, reflecting that extra‐ and intracellular freezing occur at similar temperatures, or rapid ice propagation between compartments, making them indistinguishable.

Between the phloem and the xylem lies the cambium, a secondary meristem that remains relatively understudied due to the difficulty in separating it from vascular tissues. It is often considered part of the bark or phloem. However, the cambium undergoes a period of dormancy, reflected in anatomical changes, such as reduced vacuole size due to dehydration (Rensing and Samuels 2004). It may seem obvious, but cellular damage to the cambium can simply lead to the death of the branch. Two concomitant processes allow the stem to resume growth: the reactivation of phloem allows the transport of carbohydrates, and the cambial growth for the production of new tissue, thus restoring hydraulic continuity (described above). The temporal dynamics of CH in the meristem area are thus essential to better predict freezing damage and plant recovery.

Laboratory freezing assays in different bud components have shown that isolated primordia have higher INA than whole buds (Endoh et al. 2014). In reproductive buds of angiosperms, large differences in INA among floral organs have been observed (Anderson and Ashworth 1987; Kaya and Kose 2019; Kaya et al. 2021). For instance, in *Prunus armenica* flowers, INA thresholds range from −1.9°C to −11.7°C in the receptacle, which is identified as the most ice‐susceptible organ. In contrast, the pistil, which is more tolerant, shows INA thresholds between −6.6°C and −17.0°C (Kaya and Kose 2019). Nevertheless, in intact *Pyrus pyrifolia* flowers, ice formation typically initiates in the sepals and/or the receptacle and subsequently spreads throughout the entire flower structure (Sekozawa et al. 2004). More recently, Hillmann et al. (2021) demonstrated that floral deacclimation is associated with ovary rehydration, as indicated by changes in relative water content (RWC) and tissue volume.

The determination of INA on detached or excised tissue does not account for water movement at the tissue, organ, or whole‐plant scale. To gain a deeper understanding of the factors driving ice formation and its propagation, this approach should be complemented by field‐based studies on whole plants.

## 5 Ice Nucleation Measurements Beyond the Lab

**FIGURE 2:**
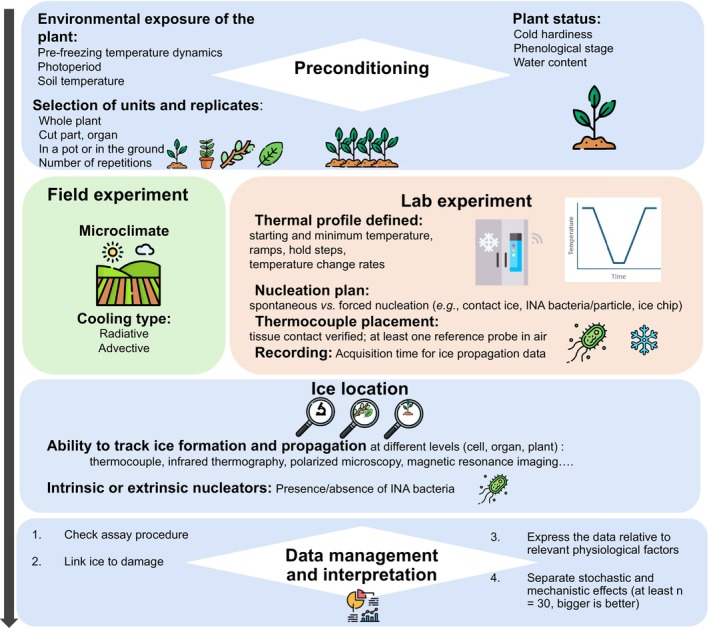
Freezing experiment workflow detailing essential checkpoints before setting a freezing experiment to avoid major pitfalls. Some of these recommendations apply to classical biological experiments, but are more specifically applicable to freezing experiments. This figure has been designed using resources from Flaticon.com.

A few points need to be taken into account when evaluating the importance of different factors related to ice nucleation in plant tissues (Figure 2). The differences between laboratory and field studies, sampling strategies, and how to avoid artificial supercooling in the laboratory are particularly important points. Our aim here is to discuss common approaches and provide a workflow for future experiments to avoid inaccurate conclusions.

### 5.1 Field‐Scale Ice Nucleation and Its Underlying Factors

Only a few studies have examined the freezing behavior of whole plants in the field, but they show that ice nucleation typically occurs in the coldest part of the plant. This is most likely the periphery of the plant, the canopy surface, or the stem base in the case of frozen soil in winter after the above‐ground organs have thawed during the day (Pramsohler et al. 2012; Charrier et al. 2017; Livingston et al. 2018). Once nucleated, a single nucleation event somewhere in the plant is sufficient to cause extracellular freezing throughout the entire plant if it is colder than 0°C and there are no internal ice barriers (Hacker and Neuner 2008). However, independent nucleation events can also be observed locally when the plant exhibits heterogeneity in temperature and INA. The freezing pattern from the base toward the distal parts commonly observed in *Picea abies* trees is probably linked to preferential nucleation sites at the position of branching and/or to an enlargement of the xylem conduits (Charrier et al. 2017). Freezing of the entire length of trees can take less than an hour (Kitaura 1967; Ashworth and Davis 1984; Pramsohler et al. 2012; Charrier et al. 2017). Generally, the rate of ice propagation is influenced by environmental conditions such as the degree of supercooling (Hacker et al. 2011), although the water status and the amount of embolism are also likely to play a significant role.

In eudicots, ice spreads longitudinally throughout the whole plant via the xylem as soon as ice comes into contact with the xylem sap (Kitaura 1967). In a second step, ice spreads radially and tangentially into other tissues, although at a lower rate (Hacker and Neuner 2007, 2008; Hacker et al. 2011; Livingston et al. 2018). In contrast, graminoids exhibit a different pattern, each leaf has its own ice nucleation event because the vessels supplying the different leaves are not connected or a thermal ice barrier by a warmer shoot (Hacker and Neuner 2008).

**FIGURE 3:**
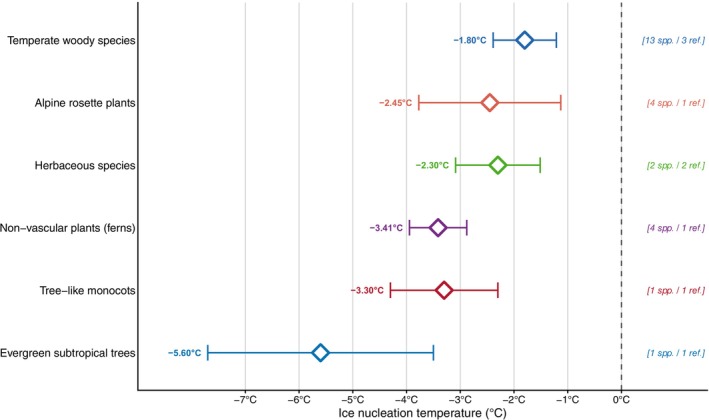
Field freezing temperatures in leaves of six different taxonomic groups. Values represent species means ± SD as reported in the original studies. The details are listed in Table S1. Group‐level statistics (mean ± propagated SD) were calculated by combining interspecific variance with the mean measurement variance of constituent species.

A comparison of field and laboratory freezing experiments on detached branches revealed that intact *Malus domestica* trees froze at −1.9°C in the field, compared with −4.6°C in the lab (Pramsohler et al. 2012). Under field conditions, ice nucleation typically occurs at mild subzero temperatures (Figure 3; Beck et al. 1982; Ashworth and Davis 1984; Ashworth, Anderson, et al. 1985; Pearce and Fuller 2001; Pramsohler et al. 2012; Stegner, Lackner, et al. 2020; Stegner et al. 2024; Fernández‐Marín et al. 2021). In trees, ice forms between −0.6°C and −2.6°C, and in leaves of herbaceous species, ferns, giant rosettes and palms not lower than −4.3°C (Figure 3). Exceptionally, lemon trees, which have sclerophyll‐like characteristics with leathery evergreen leaves, tend to freeze at lower temperatures than leaves of other tree species, between −3.5°C and −7.7°C (Stegner et al. unpublished data). It seems that species known to be more cold hardy and more frequently exposed to freezing conditions actually freeze at higher temperatures.

The main factors affecting ice nucleation temperatures in plants are the sample size, the exposure duration, and the cooling type. Differences from the field dynamics may be particularly relevant when working in the laboratory (Figure 2, Ashworth, Davis, and Anderson 1985).

1. Sample size: A logarithmic relationship between sample fresh weight (FW) and nucleation temperature was observed in peach trees, reaching a plateau above 5 g (Ashworth and Davis 1984). Consequently, small samples can artificially supercool below −10°C. In *Malus domestica* trees, ice nucleation temperature increased with increasing twig length: short twigs (1 cm) froze at −5.5°C ± 1.0°C, and long twigs (40 cm) at −2.0°C ± 0.3°C, that is, close to the field ice nucleation temperature (Pramsohler et al. 2012). It has been hypothesized that larger samples contain more ice nuclei, resulting in a higher probability of nucleation (Ashworth, Davis, and Anderson 1985).
2. Exposure duration: Longer subzero exposure increases the likelihood of ice nucleation at a given temperature. In crop seedlings held at a constant sub‐zero temperature (−5°C), the number of plants that froze increased with exposure duration (Ashworth, Davis, and Anderson 1985). This is particularly evident in ice‐susceptible plants: in *Satsuma mandarin*, exposure time significantly affected ice nucleation temperature and, consequently, freezing damage (Ebel et al. 2004).
3. Cooling type: Under natural field conditions, radiative cooling is often the dominant process during clear, calm nights. In radiative frost, plant surfaces become significantly colder than the ambient air due to longwave heat loss to the sky (Jordan and Smith 1994). By contrast, advective frost occurs when cold air masses are transported by convection (Box 1). Laboratory experiments typically simulate convective cooling in a pseudo‐equilibrium; plants remain close to or warmer than the surrounding air, which generates typical environmental conditions that fairly differ from the radiative cooling observed in many field frosts (Marcellos 1981). Creating climate chambers that simulate radiative frost in the lab on whole plants could provide key observations on ice nucleation of field conditions (Fuller and Grice 1998).

### 5.2 Avoiding Artificial Supercooling in the Laboratory

Freezing an intact mature tree is often not feasible and we have to extrapolate from experiments on excised plant parts. However, artificial supercooling must be avoided, as it affects the freezing process and damages it, consequently leading to inaccurate conclusions with respect to frost vulnerability (Arora 2018). This is particularly critical in samples where the ice nucleation temperature is close to the lethal temperature. When the CH is low, in both freezing‐tolerant and ice‐susceptible samples, artificial supercooling will lead to a significant overestimation of CH.

How to simulate a natural ice nucleation event in the lab? First, it is essential to know the realistic ice nucleation temperature of the studied species and organ in situ (Figure 2). The in situ ice nucleation temperature can be observed by using methods that detect the latent heat release of ice, such as thermocouples or infrared thermography (Ashworth and Davis 1986; Pramsohler et al. 2012; Charrier et al. 2017; Box 3). As the duration of negative temperature exposure is a critical factor for ice nucleation, the faster the temperature drops, the lower the ice nucleation temperature; To avoid artificially low ice nucleation temperatures, the cooling rate should mimic natural conditions, that is, 1–3 K h^−1^ (Levitt 1980; Ashworth, Davis, and Anderson 1985; Steffen et al. 1989; Neuner and Hacker 2012; Arora 2018). Only such moderate rates allow for dehydration dynamics as they would occur in nature (i.e., prevent artificial insufficient dehydration). In small samples, ice nucleation should be artificially induced in the range of natural ice nucleation temperatures (Figure 2) by use of biological ice nucleators or ice seeding techniques (e.g., adding a small ice crystal directly to the sample). Among the biological ice nucleators, *Pseudomonas syringae* bacteria are particularly effective. Without resorting to culturing bacteria in a laboratory, an INA formulation is commercially available as Snomax (Snomax International).

An accurate location of ice in plant tissues is essential to decipher between avoidance and tolerance strategies to prevent freezing damage (Figure 2). The temperature and location of ice nucleation within the sample indicate which areas are affected by supercooling and/or dehydration of adjacent cells and tissues that remain unfrozen (Ishikawa et al. 1997, 2015; Willick et al. 2019; Neuner, Kreische, et al. 2019). Some visualization techniques by confocal microscopy equipped with a cooling system and polarized light are powerful tools for high‐resolution detection of ice (Stegner, Wagner, and Neuner 2020; Stegner, Lackner, et al. 2020). However, the problem of sectioning frozen tissue without artifacts is a long‐standing challenge (Olien and Smith 1977) and technical improvements are still needed to observe ice without disturbing its formation, especially in vivo. Among visualization techniques, magnetic resonance imaging (MRI), by detecting water mobility, has the potential to monitor ice front propagation (Price et al. 1997; Ishikawa et al. 1997; Ide et al. 1998), but high expenses and machine time are still limiting the number of studies (Ishikawa et al. 2016).

In conclusion, it is crucial to first understand the whole plant perspective and gather knowledge about field freezing behavior, particularly when studying small plant parts or cut organs.

## 6 Conclusion and Future Direction

Ice nucleation in plants is a complex phenomenon. We emphasize that sample size and anatomical continuity play a crucial role in the initiation and propagation of ice, and fragmenting the plant material may distort the natural dynamics of freezing events. New experimental approaches that embrace interdisciplinarity are required to better reflect natural conditions of ice nucleation, including the duration of negative temperature exposure and type of frost (advective and radiative).

For perennial plants, we propose a focus on two major axes:

- Although intrinsic ice nuclei play a central role in freezing resistance mechanisms, their nature remains poorly understood: Which molecules are involved? How are intrinsic ice nuclei regulated, especially at the interface between CH and climatic conditions? How do climatic conditions and phenological transitions influence their activity and distribution within plant tissues? How are they stimulated or inhibited, from a genetic and cellular point of view? Addressing these questions will require targeted studies to understand the contribution of intrinsic ice nuclei to cold stress in plants.
- The formation of gas bubbles, a key factor in winter embolism, is linked to the ice nucleation processes. The size of these bubbles is crucial in determining embolism risk, yet the biophysical and anatomical factors involved in bubble formation remain unclear. Clarifying this process will be essential to better predict freezing‐induced hydraulic failure.

Future strategies for crop adaptation to freezing stress should focus on (1) field practices to limit INA and (2) identify genetic control of key vulnerability traits. In the field and in the case of external ice nuclei, solutions involving the application of a temporary “antinucleation” agent would help reduce the risk of freezing (delay or inhibit ice nucleation), such as hydrophobic products (Wisniewski et al. 2002). On the contrary, in the case of a major role of internal ice nuclei, agricultural solutions should instead rely on genetic selection, since spray products would have only limited effectiveness. Specifically, breeding programs should focus on minimizing the number of intrinsic ice nucleators, enhancing physical barriers against ice propagation, and optimizing phenological timing to evade critical late freezing events. Extensive research must be conducted on a wide range of plants in order to address climate and agricultural challenges while taking into account microclimatic conditions induced by the topography of the land (cold valleys, windbreaks hedges, management of cover crops, selection of species and cultivars) to gain accuracy in predicting these weather‐related risks. In summary, an integrative approach, synthesizing plant physiology, anatomy, physics and microclimate, is essential to develop practices for mitigating both radiative and advective freezing damages.

## Author Contributions

G.C. proposed the article idea. The following authors were responsible for each section: L.L. and N.D. (Section 1); C.E.M., L.L., and P.A. (Section 2); G.N. and M.S. (Section 3); A.L., G.C., K.C.‐V., N.D., and S.I. (Section 4); G.N., M.S., and N.D. (Section 5); and L.L. and N.D. (Section 6). L.L. and N.D. were responsible for coordinating the communication and planning of the manuscript. Figure 1 was created by L.L., Figure 2 by N.D., and Figure 3 by M.S. All the authors revised and gave final approval of the version to be published.

## Funding

This research was funded in whole or in part by the Austrian Science Fund (FWF) 10.55776/P34844 and 10.55776/PAT7612223 (for G.N. and M.S.), the Life program of the European Commission (Frostdefend LIFE20 CCA/GR/001747, for G.C. and N.D.), the French National Research Agency and B‐Hive engineering (Protecfroid ANR no. 15000953‐1388, for L.L., C.E.M., and G.C.), and France AgriMer (Casdar Vaccin, for G.C.).

## Supporting information


**Table S1:** Mean field ice nucleation temperatures compiled from published studies or personal data for 22 species belonging to six taxonomic groups.

## Data Availability

Data supporting Figure 3 are in the Supporting Information; additional information is available from the authors upon reasonable request.

## References

[ppl70961-bib-0001] Améglio, T. , G. Alves , M. Decourteix , et al. 2006. “Winter Biology in Walnut Tree: Freezing Tolerance by Cold Acclimation and Embolism Repair.” Acta Horticulturae 705: 241–249.

[ppl70961-bib-0002] Améglio, T. , H. Cochard , and F. Ewers . 2001. “Stem Diameter Variations and Cold Hardiness in Walnut Trees.” Journal of Experimental Botany 52: 2135–2142. 11604452 10.1093/jexbot/52.364.2135

[ppl70961-bib-0003] Anderson, J. , and D. E. G. Ashworth . 1987. “Nonbacterial Ice Nucleation in Peach Shoots.” Journal of the American Society for Horticultural Science 112: 215–218.

[ppl70961-bib-0004] Arias, N. , F. Scholz , G. Goldstein , and S. Bucci . 2017. “The Cost of Avoiding Freezing in Stems: Trade‐Off Between Xylem Resistance to Cavitation and Supercooling Capacity in Woody Plants.” Tree Physiology 37: 1251–1262. 28633378 10.1093/treephys/tpx071

[ppl70961-bib-0005] Arora, R. 2018. “Mechanism of Freeze‐Thaw Injury and Recovery: A Cool Retrospective and Warming up to New Ideas.” Plant Science 270: 301–313. 29576084 10.1016/j.plantsci.2018.03.002

[ppl70961-bib-0006] Aryal, B. , and G. Neuner . 2010. “Leaf Wettability Decreases Along an Extreme Altitudinal Gradient.” Oecologia 162, no. 1: 1–9. 19727830 10.1007/s00442-009-1437-3

[ppl70961-bib-0007] Asahina, E. 1956. “The Freezing Process of Plant Cells.” Contributions From the Institute of Low Temperature Science 10: 83–126.

[ppl70961-bib-0008] Ashworth, E. 1984. “Xylem Development in Prunus Flower Buds and the Relationship to Deep Supercooling.” Plant Physiology 74: 862–865. 16663523 10.1104/pp.74.4.862 PMC1066782

[ppl70961-bib-0009] Ashworth, E. 1992. “Formation and Spread of Ice in Plants.” Horticultural Reviews 13: 215–255.

[ppl70961-bib-0010] Ashworth, E. , J. Anderson , G. Davis , and G. Lightner . 1985. “Ice Formation in *Prunus persica* Under Field Conditions.” Journal of the American Society for Horticultural Science 110: 322–324.

[ppl70961-bib-0011] Ashworth, E. , and G. Davis . 1984. “Ice Nucleation Within Peach Trees.” Journal of the American Society for Horticultural Science 109: 198–201.

[ppl70961-bib-0012] Ashworth, E. , G. Davis , and G. Anderson . 1985. “Factors Affecting Ice Nucleation in Plant Tissues.” Plant Physiology 79: 1033–1037. 16664524 10.1104/pp.79.4.1033 PMC1075021

[ppl70961-bib-0013] Ashworth, E. , P. Echlin , R. Pearce , and T. Hayes . 1988. “Ice Formation and Tissue Response in Apple Twigs.” Plant, Cell & Environment 11: 703–710.

[ppl70961-bib-0014] Ashworth, E. , T. Willard , and S. Malone . 1992. “The Relationship Between Vascular Differentiation and the Distribution of Ice Within Forsythia Flower Buds.” Plant, Cell & Environment 15: 607–612.

[ppl70961-bib-0208] Ashworth, E. N. , and F. B. Abeles . 1984. “Freezing Behavior of Water in Small Pores and the Possible Role in the Freezing of Plant Tissues.” Plant Physiology 76, no. 1: 201–204. 16663798 10.1104/pp.76.1.201 PMC1064256

[ppl70961-bib-0015] Ashworth, E. N. , and G. A. Davis . 1986. “Ice Formation in Woody Plants Under Field Conditions.” HortScience 21, no. 5: 1233–1234.

[ppl70961-bib-0016] Atkinson, J. , B. Murray , M. Woodhouse , et al. 2013. “The Importance of Feldspar for Ice Nucleation by Mineral Dust in Mixed‐Phase Clouds.” Nature 498: 355–358. 23760484 10.1038/nature12278

[ppl70961-bib-0017] Attard, E. , H. Yang , A. Delort , et al. 2012. “Effects of Atmospheric Conditions on Ice Nucleation Activity of Pseudomonas.” Atmospheric Chemistry and Physics 12: 10667–10677.

[ppl70961-bib-0018] Beck, E. , M. Senser , R. Scheibe , H. Steiger , and P. Pongratz . 1982. “Frost Avoidance and Freezing Tolerance in Afroalpine ‘Giant Rosette’ Plants.” Plant, Cell & Environment 5: 215–222.

[ppl70961-bib-0019] Bertel, C. , J. Hacker , and G. Neuner . 2021. “Protective Role of Ice Barriers: How Reproductive Organs of Early Flowering and Mountain Plants Escape Frost Injuries.” Plants 10: 1031. 34065614 10.3390/plants10051031 PMC8161042

[ppl70961-bib-0020] Bi, Y. , B. Cao , and T. Li . 2017. “Enhanced Heterogeneous Ice Nucleation by Special Surface Geometry.” Nature Communications 8: 1–7. 10.1038/ncomms15372 PMC5442314 28513603

[ppl70961-bib-0021] Bogler, S. , and N. Borduas‐Dedekind . 2020. “Lignin's Ability to Nucleate Ice via Immersion Freezing and Its Stability Towards Physicochemical Treatments and Atmospheric Processing.” Atmospheric Chemistry and Physics 20: 14509–14522.

[ppl70961-bib-0022] Bokhorst, S. , J. Bjerke , F. Bowles , J. Melillo , T. V. Callaghan , and G. Phoenix . 2008. “Impacts of Extreme Winter Warming in the Sub‐Arctic: Growing Season Responses of Dwarf Shrub Heathland.” Global Change Biology 14: 2603–2612.

[ppl70961-bib-0023] Bose, S. , D. Pal , and P. Ariya . 2024. “On the Role of Starchy Grains in Ice Nucleation Processes.” ACS Food Science and Technology 4: 1039–1051. 38779384 10.1021/acsfoodscitech.3c00561 PMC11106773

[ppl70961-bib-0024] Brown, M. , and F. Reuter . 1974. “Freezing of Nonwoody Plant Tissues.” Cryobiology 11: 185–191. 4455465 10.1016/0011-2240(74)90092-3

[ppl70961-bib-0025] Brush, R. , M. Griffith , and A. Mlynarz . 1994. “Characterization and Quantification of Intrinsic Ice Nucleators in Winter Rye ( *Secale cereale* ) Leaves.” Plant Physiology 104: 725–735. 12232122 10.1104/pp.104.2.725 PMC159252

[ppl70961-bib-0026] Burke, M. , L. V. Gusta , H. Quamme , C. Weiser , and P. Li . 1976. “Freezing and Injury in Plants.” Annual Review of Plant Physiology 27: 507–528.

[ppl70961-bib-0027] Burke, M. , and S. Lindow . 1990. “Surface Properties and Size of the Ice Nucleation Site in Ice Nucleation Active Bacteria: Theoretical Considerations.” Cryobiology 27: 80–84.

[ppl70961-bib-0028] Chambers, R. , and H. Hale . 1932. “The Formation of Ice in Protoplasm.” Proceedings of the Royal Society of London. Series B, Containing Papers of a Biological Character 110: 336–352.

[ppl70961-bib-0029] Charra‐Vaskou, K. , E. Badel , G. Charrier , et al. 2016. “Cavitation and Water Fluxes Driven by Ice Water Potential in *Juglans regia* During Freeze–Thaw Cycles.” Journal of Experimental Botany 67: 739–750. 26585223 10.1093/jxb/erv486 PMC4737071

[ppl70961-bib-0030] Charra‐Vaskou, K. , A. Lintunen , T. Améglio , et al. 2023. “Xylem Embolism and Bubble Formation During Freezing Suggest Complex Dynamics of Pressure in *Betula pendula* Stems.” Journal of Experimental Botany 74: 5840–5853. 37463327 10.1093/jxb/erad275

[ppl70961-bib-0031] Charrier, G. , K. Charra‐Vaskou , B. Legros , T. Améglio , and S. Mayr . 2014. “Changes in Ultrasound Velocity and Attenuation Indicate Freezing of Xylem Sap.” Agricultural and Forest Meteorology 185: 20–25.

[ppl70961-bib-0032] Charrier, G. , H. Cochard , and T. Améglio . 2013. “Evaluation of the Impact of Frost Resistances on Potential Altitudinal Limit of Trees.” Tree Physiology 33: 891–902. 24052567 10.1093/treephys/tpt062

[ppl70961-bib-0033] Charrier, G. , J. Ngao , M. Saudreau , and T. Améglio . 2015. “Effects of Environmental Factors and Management Practices on Microclimate, Winter Physiology, and Frost Resistance in Trees.” Frontiers in Plant Science 6: 113228. 10.3389/fpls.2015.00259 PMC4411886 25972877

[ppl70961-bib-0034] Charrier, G. , M. Nolf , G. Leitinger , et al. 2017. “Monitoring of Freezing Dynamics in Trees: A Simple Phase Shift Causes Complexity.” Plant Physiology 173: 2196–2207. 28242655 10.1104/pp.16.01815 PMC5373037

[ppl70961-bib-0035] Charrier, G. , I. Willick , and D. Takahashi . 2023. “Cross‐Disciplinary Insights Into the Mechanisms of Plant Cold Hardiness: From Molecules to Ecosystems.” Physiologia Plantarum 175: 1–4. 10.1111/ppl.13901 37096430

[ppl70961-bib-0036] Chen, P. , M. Burke , and P. Li . 1976. “The Frost Hardiness of Several Solanum Species in Relation to the Freezing of Water, Melting Point Depression, and Tissue Water Content.” Botanical Gazette 137: 313–317.

[ppl70961-bib-0037] Cinotti, B. 1990. “La Gélivure des Chênes: Facteurs Prédisposants Individuels et Mécanique du Phénomène.” Revue Forestière Française 42: 145–147.

[ppl70961-bib-0038] Cinotti, B. 1991. “Recherche de Propriétés Intrinsèques du Bois Pouvant Expliquer la Sensibilité à la Gélivure de Quercus Petraea (Liebl) et *Q. robur* (L).” Annales des Sciences Forestières 48: 453–468.

[ppl70961-bib-0039] Cochet, N. , and P. Widehem . 2000. “Ice Crystallization by *Pseudomonas syringae* .” Applied Microbiology and Biotechnology 54: 153–161. 10968626 10.1007/s002530000377

[ppl70961-bib-0040] Conrad, P. , G. Ewing , R. Karlinsey , and V. Sadtchenko . 2005. “Ice Nucleation on BaF 2(111).” Journal of Chemical Physics 122: 064709. 15740398 10.1063/1.1844393

[ppl70961-bib-0041] Cruiziat, P. , H. Cochard , and T. Améglio . 2002. “Hydraulic Architecture of Trees: Main Concepts and Results.” Annals of Forest Science 59: 723–752.

[ppl70961-bib-0042] Davis, S. , J. Sperry , and U. Hacke . 1999. “The Relationship Between Xylem Conduit Diameter and Cavitation Caused by Freezing.” American Journal of Botany 86: 1367–1372. 10523278

[ppl70961-bib-0043] Degrandpre, E. , M. Degrandpre , B. Colman , and H. Valett . 2021. “Observations of River Solute Concentrations During Ice Formation.” ACS ES&T Water 1, no. 8: 1695–1701.

[ppl70961-bib-0044] Deville, S. 2017. “Investigating the Freezing of Colloids: Experimental Techniques to Probe Solidification Patterns, Crystal Growth, and Particle Movement.” In Freezing Colloids: Observations, Principles, Control, and Use: Applications in Materials Science, Life Science, Earth Science, Food Science, and Engineering, 47–90. Springer International Publishing.

[ppl70961-bib-0045] Diehl, K. , C. Quick , S. Matthias‐Maser , S. Mitra , and R. Jaenicke . 2001. “The Ice Nucleating Ability of Pollen: Part I: Laboratory Studies in Deposition and Condensation Freezing Modes.” Atmospheric Research 58: 75–87.

[ppl70961-bib-0046] Ebel, R. , P. Carter , W. Dozier , et al. 2004. “Pattern of Exotherm and Electrolyte Leakage Measured at High Frequency of Satsuma Mandarin Exposed to Subfreezing Temperatures.” HortScience 39: 1614–1616.

[ppl70961-bib-0047] Endoh, K. , C. Kuwabara , K. Arakawa , and S. Fujikawa . 2014. “Consideration of the Reasons Why Dormant Buds of Trees Have Evolved Extraorgan Freezing as an Adaptation for Winter Survival.” Environmental and Experimental Botany 106: 52–59.

[ppl70961-bib-0048] Fernández‐Marín, B. , M. Arzac , M. López‐Pozo , et al. 2021. “Frozen in the Dark: Interplay of Night‐Time Activity of Xanthophyll Cycle, Xylem Attributes, and Desiccation Tolerance in Fern Resistance to Winter.” Journal of Experimental Botany 72: 3168–3184. 33617637 10.1093/jxb/erab071

[ppl70961-bib-0049] Fitzner, M. , G. Sosso , S. Cox , and A. Michaelides . 2015. “The Many Faces of Heterogeneous Ice Nucleation: Interplay Between Surface Morphology and Hydrophobicity.” Journal of the American Chemical Society 137: 13658–13669. 26434775 10.1021/jacs.5b08748

[ppl70961-bib-0050] Fletcher, N. 1959. “Entropy Effect in Ice Crystal Nucleation.” Journal of Chemical Physics 30: 1476–1482.

[ppl70961-bib-0051] Fröhlich‐Nowoisky, J. , T. Hill , B. Pummer , P. Yordanova , G. Franc , and U. Pöschl . 2015. “Ice Nucleation Activity in the Widespread Soil Fungus Mortierella Alpina.” Biogeosciences 12: 1057–1071.

[ppl70961-bib-0052] Fuller, M. P. , and P. L. Grice . 1998. “A Chamber for the Simulation of Radiation Freezing of Plants.” Annals of Applied Biology 133, no. 1: 111–121.

[ppl70961-bib-0053] George, M. , M. Burke , H. Pellett , and A. Johnson . 1974. “Low Temperature Exotherms and Woody Plant Distribution1.” HortScience 9: 519–522.

[ppl70961-bib-0054] Goldstein, G. , and P. Nobel . 1991. “Changes in Osmotic Pressure and Mucilage During Low‐Temperature Acclimation of Opuntia Ficus‐Indica.” Plant Physiology 97: 954–961. 16668536 10.1104/pp.97.3.954 PMC1081109

[ppl70961-bib-0055] Gonzalez Antivilo, F. , R. C. Paz , J. Tognetti , et al. 2020. “Winter Injury to Grapevine Secondary Phloem and Cambium Impairs Budbreak, Cambium Activity, and Yield Formation.” Journal of Plant Growth Regulation 39, no. 3: 1095–1106.

[ppl70961-bib-0056] Gorb, S. , and E. V. Gorb . 2022. “Anti‐Icing Strategies of Plant Surfaces: The Ice Formation on Leaves Visualized by Cryo‐SEM Experiments.” Science of Nature 109: 1–14. 10.1007/s00114-022-01789-7 PMC8979935 35377000

[ppl70961-bib-0057] Govindarajan, A. G. , and S. E. Lindow . 1988. “Size of Bacterial Ice‐Nucleation Sites Measured In Situ by Radiation Inactivation Analysis.” Proceedings of the National Academy of Sciences 85, no. 5: 1334–1338. 10.1073/pnas.85.5.1334 PMC279765 16593912

[ppl70961-bib-0058] Gross, D. , Y. Cody , J. E. Proebsting , G. Radamaker , and R. Spotts . 1983. “Distribution, Population Dynamics, and Characteristics of Ice Nucleation‐Active Bacteria in Deciduous Fruit Tree Orchards.” Applied and Environmental Microbiology 46: 1370–1379. 16346445 10.1128/aem.46.6.1370-1379.1983 PMC239578

[ppl70961-bib-0060] Gross, D. , E. Proebsting Jr. , and P. Andrews . 1984. “The Effects of Ice Nucleation‐Active Bacteria on Temperatures of Ice Nucleation and Freeze Injury of Prunus Flower Buds at Various Stages of Development.” Journal of the American Society for Horticultural Science 109: 375–380.

[ppl70961-bib-0059] Gross, D. , E. Proebsting , and H. Maccrindle‐Zimmerman . 1988. “Development, Distribution, and Characteristics of Intrinsic, Nonbacterial Ice Nuclei in Prunus Wood.” Plant Physiology 88: 915–922. 16666404 10.1104/pp.88.3.915 PMC1055682

[ppl70961-bib-0061] Gusta, L. V. , M. Wisniewski , N. Nesbitt , and M. Gusta . 2004. “The Effect of Water, Sugars, and Proteins on the Pattern of Ice Nucleation and Propagation in Acclimated and Nonacclimated Canola Leaves.” Plant Physiology 135: 1642–1653. 15247390 10.1104/pp.103.028308 PMC519078

[ppl70961-bib-0062] Guy, C. 1990. “Cold Acclimation and Freezing Stress Tolerance: Role of Protein Metabolism.” Annual Review of Plant Physiology and Plant Molecular Biology 41: 187–223.

[ppl70961-bib-0063] Hacker, J. , U. Ladinig , J. Wagner , and G. Neuner . 2011. “Inflorescences of Alpine Cushion Plants Freeze Autonomously and May Survive Subzero Temperatures by Supercooling.” Plant Science 180: 149–156. 21151351 10.1016/j.plantsci.2010.07.013 PMC2987464

[ppl70961-bib-0064] Hacker, J. , and G. Neuner . 2007. “Ice Propagation in Plants Visualized at the Tissue Level by Infrared Differential Thermal Analysis (IDTA).” Tree Physiology 27: 1661–1670. 17938098 10.1093/treephys/27.12.1661

[ppl70961-bib-0065] Hacker, J. , and G. Neuner . 2008. “Ice Propagation in Dehardened Alpine Plant Species Studied by Infrared Differential Thermal Analysis (IDTA).” Arctic, Antarctic, and Alpine Research 40: 660–670.

[ppl70961-bib-0066] Hader, J. , T. Wright , and M. Petters . 2014. “Contribution of Pollen to Atmospheric Ice Nuclei Concentrations.” Atmospheric Chemistry and Physics 14: 5433–5449.

[ppl70961-bib-0067] Hill, T. , P. Demott , Y. Tobo , et al. 2016. “Sources of Organic Ice Nucleating Particles in Soils.” Atmospheric Chemistry and Physics 16: 7195–7211.

[ppl70961-bib-0068] Hillmann, L. , M. Elsysy , C. Goeckeritz , et al. 2021. “Preanthesis Changes in Freeze Resistance, Relative Water Content, and Ovary Growth Preempt Bud Phenology and Signify Dormancy Release of Sour Cherry Floral Buds.” Planta 254: 1–12. 34529136 10.1007/s00425-021-03722-0

[ppl70961-bib-0069] Hiraki, H. , N. Liu , J. Wang , J. Stobbs , C. Karunakaran , and K. Tanino . 2018. “Soft X‐Ray Spectromicroscopy: A Versatile Tool to Probe Pristine Plant Cell Walls.” Microscopy and Microanalysis 24: 356–357.

[ppl70961-bib-0070] Hiranuma, N. , O. Möhler , K. Yamashita , et al. 2015. “Ice Nucleation by Cellulose and Its Potential Contribution to Ice Formation in Clouds.” Nature Geoscience 8: 273–277.

[ppl70961-bib-0071] Hoose, C. , and O. Möhler . 2012. “Heterogeneous Ice Nucleation on Atmospheric Aerosols: A Review of Results From Laboratory Experiments.” Atmospheric Chemistry and Physics 12, no. 20: 9817–9854.

[ppl70961-bib-0072] Ide, H. , W. S. Price , Y. Arata , and M. Ishikawa . 1998. “Freezing Behaviors in Leaf Buds of Cold‐Hardy Conifers Visualized by NMR Microscopy.” Tree Physiology 18, no. 7: 451–458. 12651356 10.1093/treephys/18.7.451

[ppl70961-bib-0073] Ingram, S. , B. Reischl , T. Vesala , and H. Vehkamäki . 2024. “Ruptures of Mixed Lipid Monolayers Under Tension and Supercooling: Implications for Nanobubbles in Plants.” Nanoscale Advances 6: 3775–3784. 39050947 10.1039/d4na00316k PMC11265596

[ppl70961-bib-0074] Ingram, S. , A. Zanetti , L. Mustonen , A. A. Piedehierro , A. Laaksonen , and A. Lintunen . 2025. “Toward Understanding Apoplastic Freezing Under Negative Pressure.” New Phytologist 248, no. 3: 1245–1254. 40937487 10.1111/nph.70538 PMC12489277

[ppl70961-bib-0075] Ishikawa, M. 2014. “Ice Nucleation Activity in Plant Tissues.” Cryobiology and Cryotechnology 60: 79–88.

[ppl70961-bib-0076] Ishikawa, M. , H. Ide , T. Tsujii , et al. 2022. “Preferential Freezing Avoidance Localised in Anthers and Embryo Sacs in Wintering Daphne Kamtschatica Var. Jezoensis Flower Buds Visualised by Magnetic Resonance Imaging.” Plant, Cell & Environment 45, no. 7: 2109–2125. 10.1111/pce.14255 34985134

[ppl70961-bib-0077] Ishikawa, M. , H. Ide , H. Yamazaki , et al. 2016. “Freezing Behaviours in Wintering *Cornus florida* Flower Bud Tissues Revisited Using MRI.” Plant, Cell & Environment 39: 2663–2675. 10.1111/pce.12813 27497429

[ppl70961-bib-0078] Ishikawa, M. , M. Ishikawa , T. Toyomasu , T. Aoki , and W. Price . 2015. “Ice Nucleation Activity in Various Tissues of Rhododendron Flower Buds: Their Relevance to Extraorgan Freezing.” Frontiers in Plant Science 6: 1–12. 25859249 10.3389/fpls.2015.00149 PMC4373250

[ppl70961-bib-0079] Ishikawa, M. , W. Price , H. Ide , and Y. Arata . 1997. “Visualization of Freezing Behaviors in Leaf and Flower Buds of Full‐Moon Maple by Nuclear Magnetic Resonance Microscopy.” Plant Physiology 115: 1515–1524. 12223878 10.1104/pp.115.4.1515 PMC158617

[ppl70961-bib-0080] Ishikawa, M. , and A. Sakai . 1981. “Freezing Avoidance Mechanisms by Supercooling in Some Rhododendron Flower Buds With Reference to Water Relations.” Plant and Cell Physiology 22: 953–967.

[ppl70961-bib-0081] Ishikawa, M. , and A. Sakai . 1982. “Characteristics of Freezing Avoidance in Comparison With Freezing Tolerance: A Demonstration of Extraorgan Freezing.” Plant Cold Hardiness and Freezing Stress 2: 325–340.

[ppl70961-bib-0082] Ishikawa, M. , and A. Sakai . 1985. “Extraorgan Freezing in Wintering Flower Buds of *Cornus officinalis* Sieb. et Zucc.” Plant, Cell & Environment 8: 333–338.

[ppl70961-bib-0083] Ishikawa, M. , H. Yamazaki , T. Kishimoto , et al. 2018. “Ice Nucleation Activity in Plants: The Distribution, Characterization, and Their Roles in Cold Hardiness Mechanisms.” In Survival Strategies in Extreme Cold and Desiccation, 99–115. Springer. 10.1007/978-981-13-1244-1_6 30288706

[ppl70961-bib-0084] Jochen, S. H. , S. Espino , D. Romo , et al. 2017. “Xylem Surfactants Introduce a New Element to the Cohesion‐Tension Theory.” Plant Physiology 173: 1177–1196. 27927981 10.1104/pp.16.01039 PMC5291718

[ppl70961-bib-0085] Joly, M. , E. Attard , M. Sancelme , et al. 2013. “Ice Nucleation Activity of Bacteria Isolated From Cloud Water.” Atmospheric Environment 70: 392–400.

[ppl70961-bib-0086] Jones, K. , B. McKersie , and J. Paroschy . 2000. “Prevention of Ice Propagation by Permeability Barriers in Bud Axes of *Vitis vinifera* .” Canadian Journal of Botany 78: 3–9.

[ppl70961-bib-0087] Jordan, D. , and W. Smith . 1994. “Energy Balance Analysis of Nighttime Leaf Temperatures and Frost Formation in a Subalpine Environment.” Agricultural and Forest Meteorology 71: 359–372.

[ppl70961-bib-0088] Kaku, S. , and M. Iwaya . 1979. “Deep Supercooling in Xylems and Ecological Distribution in the Genera Ilex, Viburnum and Quercus in Japan.” Oikos 33: 402–411.

[ppl70961-bib-0089] Kashchiev, D. 2000. “11 Nucleation Rate.” In Nucleation: Basic Theory With Applications. Butterworth‐Heinemann.

[ppl70961-bib-0090] Kaya, O. , and C. Kose . 2019. “Cell Death Point in Flower Organs of Some Apricot ( *Prunus armeniaca* L.) Cultivars at Subzero Temperatures.” Scientia Horticulturae 249: 299–305.

[ppl70961-bib-0091] Kaya, O. , C. Kose , A. Esitken , et al. 2021. “Frost Tolerance in Apricot ( *Prunus armeniaca* L.) Receptacle and Pistil Organs: How Is the Relationship Among Amino Acids, Minerals, and Cell Death Points?” International Journal of Biometeorology 65: 2157–2170. 34324064 10.1007/s00484-021-02178-x

[ppl70961-bib-0092] Kinney, N. , C. Hepburn , M. Gibson , D. Ballesteros , and T. Whale . 2024. “High Interspecific Variability Indicates Pollen Ice Nucleators Are Incidental.” EGUsphere: 1–22.

[ppl70961-bib-0093] Kishimoto, T. , Y. Sekozawa , H. Yamazaki , H. Murakawa , K. Kuchitsu , and M. Ishikawa . 2014. “Seasonal Changes in Ice Nucleation Activity in Blueberry Stems and Effects of Cold Treatments In Vitro.” Environmental and Experimental Botany 106: 13–23.

[ppl70961-bib-0094] Kishimoto, T. , H. Yamazaki , A. Saruwatari , et al. 2014. “High Ice Nucleation Activity Located in Blueberry Stem Bark Is Linked to Primary Freeze Initiation and Adaptive Freezing Behaviour of the Bark.” AoB Plants 6: plu044. 25082142 10.1093/aobpla/plu044 PMC4224666

[ppl70961-bib-0095] Kitaura, K. 1967. “Supercooling and Ice Formation in Mulberry Trees.” Cellular Injury and Resistance in Freezing Organisms: Proceedings 2: 143–156.

[ppl70961-bib-0096] Knopf, D. , P. Alpert , B. Wang , and J. Aller . 2011. “Stimulation of Ice Nucleation by Marine Diatoms.” Nature Geoscience 4: 88–90.

[ppl70961-bib-0097] Koop, T. , B. Luo , A. Tsias , and T. Peter . 2000. “Water Activity as the Determinant for Homogeneous Ice Nucleation in Aqueous Solutions.” Nature 406: 611–614. 10949298 10.1038/35020537

[ppl70961-bib-0098] Körner, C. 2021. Alpine Plant Life: Functional Plant Ecology of High Mountain Ecosystems, 1–500. Springer International Publishing.

[ppl70961-bib-0099] Krog, J. , K. Zachariassen , B. Larsen , and O. Smidsrod . 1979. “Thermal Buffering in Afro‐Alpine Plants due to Nucleating Agent‐Inducing Water Freezing.” Nature 282: 300–301.

[ppl70961-bib-0100] Kuprian, E. , V. Briceño , J. Wagner , and G. Neuner . 2014. “Ice Barriers Promote Supercooling and Prevent Frost Injury in Reproductive Buds, Flowers and Fruits of Alpine Dwarf Shrubs Throughout the Summer.” Environmental and Experimental Botany 106: 4–12. 25284910 10.1016/j.envexpbot.2014.01.011 PMC4104041

[ppl70961-bib-0101] Kuprian, E. , S. Koch , C. Munkler , et al. 2018. “Does Winter Desiccation Account for Seasonal Increases in Supercooling Capacity of Norway Spruce Bud Primordia?” Tree Physiology 38: 591–601. 29182788 10.1093/treephys/tpx142 PMC5895075

[ppl70961-bib-0102] Kuprian, E. , C. Munkler , A. Resnyak , et al. 2017. “Complex Bud Architecture and Cell‐Specific Chemical Patterns Enable Supercooling of *Picea abies* Bud Primordia.” Plant, Cell & Environment 40: 3101–3112. 10.1111/pce.13078 PMC5725666 28960368

[ppl70961-bib-0103] Kuprian, E. , T. Tuong , K. Pfaller , J. Wagner , D. Livingston , and G. Neuner . 2016. “Persistent Supercooling of Reproductive Shoots Is Enabled by Structural Ice Barriers Being Active Despite an Intact Xylem Connection.” PLoS One 11: 1–15. 10.1371/journal.pone.0163160 PMC5025027 27632365

[ppl70961-bib-0104] Ladinig, U. , J. Hacker , G. Neuner , and J. Wagner . 2013. “How Endangered Is Sexual Reproduction of High‐Mountain Plants by Summer Frosts? Frost Resistance, Frequency of Frost Events and Risk Assessment.” Oecologia 171: 743–760. 23386042 10.1007/s00442-012-2581-8 PMC3599211

[ppl70961-bib-0105] Levitt, J. 1980. Responses of Plants to Environmental Stress. Volume 1: Chilling, Freezing, and High Temperature Stresses. Academic Press.

[ppl70961-bib-0106] Lindow, S. 2023. “History of Discovery and Environmental Role of Ice Nucleating Bacteria.” Phytopathology 113: 605–615. 36122194 10.1094/PHYTO-07-22-0256-IA

[ppl70961-bib-0107] Lintunen, A. , L. Lindfors , P. Kolari , E. Juurola , E. Nikinmaa , and T. Hölttä . 2014. “Bursts of CO_2_ Released During Freezing Offer a New Perspective on Avoidance of Winter Embolism in Trees.” Annals of Botany 114: 1711–1718. 25252688 10.1093/aob/mcu190 PMC4649691

[ppl70961-bib-0108] Lintunen, A. , L. Lindfors , E. Nikinmaa , and T. Hölttä . 2017. “Xylem Diameter Changes During Osmotic Stress, Desiccation and Freezing in Pinus Sylvestris and *Populus tremula* .” Tree Physiology 37: 491–500. 27998974 10.1093/treephys/tpw114

[ppl70961-bib-0109] Lintunen, A. , A. Losso , J. Aalto , T. Chan , T. Hölttä , and S. Mayr . 2020. “Propagating Ice Front Induces Gas Bursts and Ultrasonic Acoustic Emissions From Freezing Xylem.” Tree Physiology 40: 170–182. 31860711 10.1093/treephys/tpz123

[ppl70961-bib-0110] Lintunen, A. , S. Mayr , Y. Salmon , H. Cochard , and T. Hölttä . 2018. “Drivers of Apoplastic Freezing in Gymnosperm and Angiosperm Branches.” Ecology and Evolution 8: 333–343. 29321875 10.1002/ece3.3665 PMC5756836

[ppl70961-bib-0111] Lintunen, A. , T. Paljakka , A. Riikonen , et al. 2015. “Irreversible Diameter Change of Wood Segments Correlates With Other Methods for Estimating Frost Tolerance of Living Cells in Freeze‐Thaw Experiment: A Case Study With Seven Urban Tree Species in Helsinki.” Annals of Forest Science 72: 1089–1098.

[ppl70961-bib-0112] Lintunen, A. , Y. Salmon , T. Hölttä , and H. Suhonen . 2022. “Inspection of Gas Bubbles in Frozen *Betula pendula* Xylem With Micro‐CT: Conduit Size, Water Status and Bark Permeability Affect Bubble Characteristics.” Physiologia Plantarum 174: e13749.

[ppl70961-bib-0113] Livingston, D. , A. Bertrand , M. Wisniewski , et al. 2021. “Factors Contributing to Ice Nucleation and Sequential Freezing of Leaves in Wheat.” Planta 253: 1–15. 10.1007/s00425-021-03637-w PMC8137482 34014374

[ppl70961-bib-0114] Livingston, D. , T. Tuong , J. Murphy , L. V. Gusta , I. Willick , and M. Wisniewski . 2018. “High‐Definition Infrared Thermography of Ice Nucleation and Propagation in Wheat Under Natural Frost Conditions and Controlled Freezing.” Planta 247: 791–806. 29224121 10.1007/s00425-017-2823-4 PMC5856896

[ppl70961-bib-0115] Maeda, N. 2021. “Brief Overview of Ice Nucleation.” Molecules 26: 392. 33451150 10.3390/molecules26020392 PMC7828621

[ppl70961-bib-0116] Maki, L. , E. Gaylan , M. Chang‐Chien , and D. Caldwell . 1974. “Ice Nucleation Induced by *Pseudomonas syringae* .” Applied Microbiology 28: 456–459. 4371331 10.1128/am.28.3.456-459.1974 PMC186742

[ppl70961-bib-0117] Marcellos, H. 1981. “A Plant Freezing Chamber With Radiative and Convective Energy Exchange.” Journal of Agricultural Engineering Research 26: 403–408.

[ppl70961-bib-0118] Mayr, S. , and K. Charra‐Vaskou . 2007. “Winter at the Alpine Timberline Causes Complex Within‐Tree Patterns of Water Potential and Embolism in *Picea abies* .” Physiologia Plantarum 131: 131–139. 18251931 10.1111/j.1399-3054.2007.00942.x

[ppl70961-bib-0119] Mayr, S. , P. Schmid , B. Beikircher , F. Feng , and E. Badel . 2020. “Die Hard: Timberline Conifers Survive Annual Winter Embolism.” New Phytologist 226: 13–20. 31677276 10.1111/nph.16304 PMC7065000

[ppl70961-bib-0120] Mayr, S. , and J. Sperry . 2010. “Freeze‐Thaw‐Induced Embolism in *Pinus contorta* : Centrifuge Experiments Validate the ‘Thaw‐Expansion Hypothesis’ but Conflict With Ultrasonic Emission Data.” New Phytologist 185: 1016–1024. 20028475 10.1111/j.1469-8137.2009.03133.x

[ppl70961-bib-0121] Mayr, S. , G. Wieser , and H. Bauer . 2006. “Xylem Temperatures During Winter in Conifers at the Alpine Timberline.” Agricultural and Forest Meteorology 137: 81–88.

[ppl70961-bib-0122] Mazur, P. 1969. “Freezing Injury in Plants.” Annual Review of Plant Physiology 20: 419–448.

[ppl70961-bib-0123] McCully, M. , M. Canny , and C. Huang . 2004. “The Management of Extracellular Ice by Petioles of Frost‐Resistant Herbaceous Plants.” Annals of Botany 94: 665–674. 15355865 10.1093/aob/mch191 PMC4242212

[ppl70961-bib-0124] McLeester, R. , C. Weiser , and T. Hall . 1969. “Multiple Freezing Points as a Test for Viability of Plant Stems in the Determination of Frost Hardiness.” Plant Physiology 44: 37–44. 16657031 10.1104/pp.44.1.37 PMC396035

[ppl70961-bib-0125] Morris, C. , C. Glaux , X. Latour , L. Gardan , R. Samson , and M. Pitrat . 2000. “The Relationship of Host Range, Physiology, and Genotype to Virulence on Cantaloupe in *Pseudomonas syringae* From Cantaloupe Blight Epidemics in France.” Phytopathology 90: 636–646. 18944544 10.1094/PHYTO.2000.90.6.636

[ppl70961-bib-0126] Murray, B. , D. O'sullivan , J. Atkinson , and M. Webb . 2012. “Ice Nucleation by Particles Immersed in Supercooled Cloud Droplets.” Chemical Society Reviews 41: 6519–6554. 22932664 10.1039/c2cs35200a

[ppl70961-bib-0127] Neuner, G. , and B. Beikircher . 2010. “Critically Reduced Frost Resistance of *Picea abies* During Sprouting Could Be Linked to Cytological Changes.” Protoplasma 243: 145–152. 19533300 10.1007/s00709-009-0052-9

[ppl70961-bib-0128] Neuner, G. , and J. Hacker . 2012. “Ice Formation and Propagation in Alpine Plants.” In Plants in Alpine Regions: Cell Physiology of Adaption and Survival Strategies, edited by C. Lütz , 163–174. Springer Vienna.

[ppl70961-bib-0129] Neuner, G. , B. Huber , A. Plangger , J. Pohlin , and J. Walde . 2020. “Low Temperatures at Higher Elevations Require Plants to Exhibit Increased Freezing Resistance Throughout the Summer Months.” Environmental and Experimental Botany 169: 103882.

[ppl70961-bib-0130] Neuner, G. , B. Kreische , D. Kaplenig , K. Monitzer , and R. Miller . 2019. “Deep Supercooling Enabled by Surface Impregnation With Lipophilic Substances Explains the Survival of Overwintering Buds at Extreme Freezing.” Plant, Cell & Environment 42: 2065–2074. 10.1111/pce.13545 PMC6618773 30827059

[ppl70961-bib-0131] Neuner, G. , K. Monitzer , D. Kaplenig , and J. Ingruber . 2019. “Frost Survival Mechanism of Vegetative Buds in Temperate Trees: Deep Supercooling and Extraorgan Freezing vs. Ice Tolerance.” Frontiers in Plant Science 10: 1–13. 31143193 10.3389/fpls.2019.00537 PMC6521125

[ppl70961-bib-0132] Neuner, G. , B. Xu , and J. Hacker . 2010. “Velocity and Pattern of Ice Propagation and Deep Supercooling in Woody Stems of *Castanea sativa* , Morus Nigra and *Quercus robur* Measured by IDTA.” Tree Physiology 30: 1037–1045. 20616300 10.1093/treephys/tpq059

[ppl70961-bib-0133] Ögren, E. 1999. “Fall Frost Resistance in Willows Used for Biomass Production. II. Predictive Relationships With Sugar Concentration and Dry Matter Content.” Tree Physiology 19: 755–760. 12651315 10.1093/treephys/19.11.755

[ppl70961-bib-0134] Olien, A. C. , and M. Smith . 1977. “Ice Adhesions in Relation to Freeze Stress.” Plant Physiology 60: 499–503. 16660124 10.1104/pp.60.4.499 PMC542650

[ppl70961-bib-0135] Olien, C. 1971. “A Comparison of Desiccation and Freezing as Stress Vectors.” Cryobiology 8: 244–248. 5570408 10.1016/0011-2240(71)90046-0

[ppl70961-bib-0136] Olien, C. 1973. “Thermodynamic Components of Freezing Stress.” Journal of Theoretical Biology 39: 201–210. 4741714 10.1016/0022-5193(73)90217-8

[ppl70961-bib-0137] Olien, C. 1974. “Energies of Freezing and Frost Desiccation.” Plant Physiology 53: 764–767. 16658785 10.1104/pp.53.5.764 PMC541441

[ppl70961-bib-0138] Palta, J. , J. Levitt , E. Stadelmann , and M. Burke . 1977. “Dehydration of Onion Cells: A Comparison of Freezing vs. Desiccation and Living vs. Dead Cells.” Physiologia Plantarum 41: 273–279.

[ppl70961-bib-0139] Pandey, R. , K. Usui , R. Livingstone , et al. 2016. “Ice‐Nucleating Bacteria Control the Order and Dynamics of Interfacial Water.” Science Advances 2, no. 4: e1501630. 27152346 10.1126/sciadv.1501630 PMC4846457

[ppl70961-bib-0140] Parker, J. 1963. “Cold Resistance in Woody Plants.” Botanical Review 29: 123–201.

[ppl70961-bib-0141] Pearce, R. 2001. “Plant Freezing and Damage.” Annals of Botany 87: 417–424.

[ppl70961-bib-0142] Pearce, R. , and M. Fuller . 2001. “Freezing of Barley Studied by Infrared Video Thermography.” Plant Physiology 125: 227–240. 11154332 10.1104/pp.125.1.227 PMC61005

[ppl70961-bib-0143] Perry, K. B. 1994. Freeze/Frost Protection for Horticultural Crops. North Carolina State University Cooperative Extension. Horticulture.

[ppl70961-bib-0144] Philip, B. , A. Kiss , and R. Lee . 2011. “The Protective Role of Aquaporins in the Freeze‐Tolerant Insect *Eurosta solidaginis* : Functional Characterization and Tissue Abundance of EsAQP1.” Journal of Experimental Biology 214: 848–857. 21307072 10.1242/jeb.051276

[ppl70961-bib-0145] Pittermann, J. , and J. Sperry . 2003. “Tracheid Diameter Is the Key Trait Determining the Extent of Freezing‐Induced Embolism in Conifers.” Tree Physiology 23: 907–914. 14532014 10.1093/treephys/23.13.907

[ppl70961-bib-0146] Pittermann, J. , and J. Sperry . 2006. “Analysis of Freeze‐Thaw Embolism in Conifers. The Interaction Between Cavitation Pressure and Tracheid Size.” Plant Physiology 140: 374–382. 16377751 10.1104/pp.105.067900 PMC1326058

[ppl70961-bib-0147] Pouleur, S. , C. Richard , J. Martin , and H. Antoun . 1992. “Ice Nucleation Activity in Fusarium Acuminatum and Fusarium Avenaceum.” Applied and Environmental Microbiology 58: 2960–2964. 16348770 10.1128/aem.58.9.2960-2964.1992 PMC183033

[ppl70961-bib-0148] Pramsohler, M. , J. Hacker , G. Neuner , and M. Ball . 2012. “Freezing Pattern and Frost Killing Temperature of Apple ( *Malus domestica* ) Wood Under Controlled Conditions and in Nature.” Tree Physiology 32: 819–828. 22628198 10.1093/treephys/tps046

[ppl70961-bib-0149] Pramsohler, M. , and G. Neuner . 2013. “Dehydration and Osmotic Adjustment in Apple Stem Tissue During Winter as It Relates to the Frost Resistance of Buds.” Tree Physiology 33: 807–816. 23939553 10.1093/treephys/tpt057

[ppl70961-bib-0150] Price, W. S. , H. Ide , Y. Arata , and M. Ishikawa . 1997. “Visualisation of Freezing Behaviours in Flower Bud Tissues of Cold‐Hardy *Rhododendron japonicum* by Nuclear Magnetic Resonance Micro‐Imaging.” Australian Journal of Plant Physiology 24, no. 5: 599–605.

[ppl70961-bib-0151] Pummer, B. , H. Bauer , J. Bernardi , S. Bleicher , and H. Grothe . 2012. “Suspendable Macromolecules Are Responsible for Ice Nucleation Activity of Birch and Conifer Pollen.” Atmospheric Chemistry and Physics 12: 2541–2550.

[ppl70961-bib-0152] Rahman, A. , Y. Kawamura , M. Maeshima , A. Rahman , and M. Uemura . 2020. “Plasma Membrane Aquaporin Members PIPs Act in Concert to Regulate Cold Acclimation and Freezing Tolerance Responses in *Arabidopsis thaliana* .” Plant & Cell Physiology 61: 787–802. 31999343 10.1093/pcp/pcaa005

[ppl70961-bib-0153] Rajashekar, C. , P. Li , and J. V. Carter . 1983. “Frost Injury and Heterogeneous Ice Nucleation in Leaves of Tuber‐Bearing Solanum Species.” Plant Physiology 71: 749–755. 16662901 10.1104/pp.71.4.749 PMC1066116

[ppl70961-bib-0154] Rajashekar, C. , M. Westwood , and M. Burke . 1982. “Deep Supercooling and Cold Hardiness in Genus *Pyrus* [Pear Species].” Journal of the American Society for Horticultural Science 107: 968–972.

[ppl70961-bib-0155] Rensing, K. , and A. Samuels . 2004. “Cellular Changes Associated With Rest and Quiescence in Winter‐Dormant Vascular Cambium of *Pinus contorta* .” Trees‐Structure and Function 18: 373–380.

[ppl70961-bib-0156] Ristic, Z. , and E. Ashworth . 1993. “Changes in Leaf Ultrastructure and Carbohydrates in *Arabidopsis thaliana* L. (Heyn) cv. Columbia During Rapid Cold Acclimation.” Protoplasma 172: 111–123.

[ppl70961-bib-0157] Rodrigo, J. 2000. “Spring Frosts in Deciduous Fruit Trees. Morphological Damage and Flower Hardiness.” Scientia Horticulturae 85: 155–173.

[ppl70961-bib-0207] Roos, I. M. , and M. J. Hattingh . 1983. “Scanning Electron Microscopy of *Pseudomonas syringae* Pv, *Morsprunorum* on Sweet Cherry Leaves.” Journal of Phytopathology 108, no. 1: 18–25.

[ppl70961-bib-0158] Saint‐Michel, B. , M. Georgelin , S. Deville , and A. Pocheau . 2017. “Interaction of Multiple Particles With a Solidification Front: From Compacted Particle Layer to Particle Trapping.” Langmuir 33: 5617–5627. 28505455 10.1021/acs.langmuir.7b00472

[ppl70961-bib-0159] Sakai, A. 1979. “Deep Supercooling of Winter Flower Buds of *Cornus florida* L. 1.” HortScience 14: 69–70.

[ppl70961-bib-0160] Sakai, A. , and W. Larcher . 1987. “Low Temperature and Frost as Environmental Factors.” In Frost Survival of Plants: Responses and Adaptation to Freezing Stress, 1–20. Springer Berlin Heidelberg.

[ppl70961-bib-0161] Schenk, H. J. , K. Steppe , and S. Jansen . 2015. “Nanobubbles: A New Paradigm for Air‐Seeding in Xylem.” Trends in Plant Science 20: 199–205. 25680733 10.1016/j.tplants.2015.01.008

[ppl70961-bib-0162] Sekozawa, Y. , S. Sugaya , and H. Gemma . 2004. “Observations of Ice Nucleation and Propagation in Flowers of Japanese Pear ( *Pyrus pyrifolia* Nakai) Using Infrared Video Thermography.” Journal of the Japanese Society for Horticultural Science 73: 1–6.

[ppl70961-bib-0163] Sevanto, S. , H. N. Michele , and M. Ball . 2012. “Freeze/Thaw‐Induced Embolism: Probability of Critical Bubble Formation Depends on Speed of Ice Formation.” Frontiers in Plant Science 3: 23604. 10.3389/fpls.2012.00107 PMC3368182 22685446

[ppl70961-bib-0164] Sevanto, S. , T. Suni , J. Pumpanen , et al. 2006. “Wintertime Photosynthesis and Water Uptake in a Boreal Forest.” Tree Physiology 26: 749–757. 16510390 10.1093/treephys/26.6.749

[ppl70961-bib-0165] Siminovitch, D. , and G. Scarth . 1938. “A Study of the Mechanism of Frost Injury to Plants.” Canadian Journal of Research 16c: 467–481.

[ppl70961-bib-0209] Simpson, N. , S. Orr , S. Sabour , et al. 2022. ICSM CHC White Paper II: Impacts, Vulnerability, and Understanding Risks of Climate Change for Culture and Heritage: Contribution of Impacts Group II to the International CoSponsored Meeting on Culture, Heritage and Climate Change. ICOMOS & ICSM CHC.

[ppl70961-bib-0166] Single, W. V. , and H. Marcellos . 1981. “Ice Formation and Freezing Injury in Actively Growing Cereals (Wheat).” In Analysis and Improvement of Plant Cold Hardiness. CRC Press.

[ppl70961-bib-0167] Sklenář, P. 2017. “Seasonal Variation of Freezing Resistance Mechanisms in North‐Temperate Alpine Plants.” Alpine Botany 127: 31–39.

[ppl70961-bib-0168] Snyder, R. L. , and J. P. Melo‐Abreu . 2005. Frost Protection: Fundamentals, Practice and Economics. Vol. 1. FAO.

[ppl70961-bib-0169] Sperry, J. , and J. Sullivan . 1992. “Xylem Embolism in Response to Freeze‐Thaw Cycles and Water Stress in Ring‐Porous, Diffuse‐Porous, and Conifer Species.” Plant Physiology 100: 605–613. 16653035 10.1104/pp.100.2.605 PMC1075601

[ppl70961-bib-0170] Steffen, K. , R. Arora , and J. Palta . 1989. “Relative Sensitivity of Photosynthesis and Respiration to Freeze‐Thaw Stress in Herbaceous Species: Importance of Realistic Freeze‐Thaw Protocols.” Plant Physiology 89: 1372–1379. 16666712 10.1104/pp.89.4.1372 PMC1056024

[ppl70961-bib-0171] Stegner, M. , B. Lackner , T. Schäfernolte , et al. 2020. “Winter Nights During Summer Time: Stress Physiological Response to Ice and the Facilitation of Freezing Cytorrhysis by Elastic Cell Wall Components in the Leaves of a Nival Species.” International Journal of Molecular Sciences 21: 7042. 32987913 10.3390/ijms21197042 PMC7582304

[ppl70961-bib-0172] Stegner, M. , A. Strasser , and G. Neuner . 2024. “Supercooling Cells of Frost Hardy Palm Leaves: Quantified Percentage of Frozen Water and Displacement From Thermodynamic Equilibrium.” Environmental and Experimental Botany 226: 105895.

[ppl70961-bib-0173] Stegner, M. , J. Wagner , and G. Neuner . 2020. “Ice Accommodation in Plant Tissues Pinpointed by Cryo‐Microscopy in Reflected‐Polarised‐Light.” Plant Methods 16: 73. 32477423 10.1186/s13007-020-00617-1 PMC7240938

[ppl70961-bib-0174] Steinke, I. , N. Hiranuma , R. Funk , et al. 2020. “Complex Plant‐Derived Organic Aerosol as Ice‐Nucleating Particles – More Than the Sums of Their Parts?” Atmospheric Chemistry and Physics 20: 11387–11397.

[ppl70961-bib-0175] Steponkus, P. , and M. Webb . 1992. “Freeze‐Induced Dehydration and Membrane Destabilization in Plants.” In Water and Life, 338–362. Springer Berlin Heidelberg.

[ppl70961-bib-0176] Stuckey, I. , and O. Curtis . 1938. “Ice Formation and the Death of Plant Cells by Freezing.” Plant Physiology 13: 815–833. 16653527 10.1104/pp.13.4.815 PMC439438

[ppl70961-bib-0177] Takeuchi, M. , and J. Kasuga . 2018. “Bark Cells and Xylem Cells in Japanese White Birch Twigs Initiate Deacclimation at Different Temperatures.” Cryobiology 80: 96–100. 29169970 10.1016/j.cryobiol.2017.11.007

[ppl70961-bib-0178] Tong, H. , Q. Hu , L. Zhu , and X. Dong . 2019. “Prokaryotic Aquaporins.” Cells 8: 1316. 31653102 10.3390/cells8111316 PMC6912568

[ppl70961-bib-0179] Towill, L. , and P. Mazur . 1976. “Osmotic Shrinkage as a Factor in Freezing Injury in Plant Tissue Cultures.” Plant Physiology 57: 290–296. 16659469 10.1104/pp.57.2.290 PMC542010

[ppl70961-bib-0180] Uemura, M. , Y. Tominaga , C. Nakagawara , S. Shigematsu , A. Minami , and Y. Kawamura . 2006. “Responses of the Plasma Membrane to Low Temperatures.” Physiologia Plantarum 126: 81–89.

[ppl70961-bib-0181] Upper, C. , and G. Vali . 1995. “The Discovery of Bacterial Ice Nucleation and Its Role in the Injury of Plants by Frost.” In Biological Ice Nucleation and Its Applications, 29–39. APS Press.

[ppl70961-bib-0182] Utsumi, Y. , Y. Sano , S. Fujikawa , R. Funada , and J. Ohtani . 1998. “Visualization of Cavitated Vessels in Winter and Refilled Vessels in Spring in Diffuse‐Porous Trees by Cryo‐Scanning Electron Microscopy.” Plant Physiology 117: 1463–1471. 9701601 10.1104/pp.117.4.1463 PMC34909

[ppl70961-bib-0183] Vali, G. 1995. “Principles of Ice Nucleation.” In Biological Ice Nucleation and Its Applications, edited by R. E. Lee Jr. , G. J. Warren , and L. V. Gusta , 1–28. APS Press.

[ppl70961-bib-0184] Vali, G. , P. DeMott , O. Möhler , and T. Whale . 2015. “Technical Note: A Proposal for Ice Nucleation Terminology.” Atmospheric Chemistry and Physics 15: 10263–10270.

[ppl70961-bib-0185] Venn, S. , J. Morgan , and J. Lord . 2013. “Foliar Freezing Resistance of Australian Alpine Plants Over the Growing Season.” Austral Ecology 38: 152–161.

[ppl70961-bib-0186] Villouta, C. , B. Workmaster , J. Bolivar‐Medina , S. Sinclair , and A. Atucha . 2020. “Freezing Stress Survival Mechanisms in *Vaccinium macrocarpon* Ait. Terminal Buds.” Tree Physiology 40: 841–855. 32163157 10.1093/treephys/tpaa028 PMC8493662

[ppl70961-bib-0187] Villouta, C. , B. Workmaster , D. Livingston , and A. Atucha . 2022. “Acquisition of Freezing Tolerance in *Vaccinium macrocarpon* Ait. Is a Multi‐Factor Process Involving the Presence of an Ice Barrier at the Bud Base.” Frontiers in Plant Science 13: 891488. 35599888 10.3389/fpls.2022.891488 PMC9115472

[ppl70961-bib-0188] Wang, K. , M. Wu , and R. Zhang . 2016. “Water and Solute Fluxes in Soils Undergoing Freezing and Thawing.” Soil Science 181: 193–201.

[ppl70961-bib-0189] Ward, P. , and P. DeMott . 1989. “Preliminary Experimental Evaluation of Snomax (TM) Snow Inducer, Nucleus *Pseudomonas syringae* , as an Artificial Ice Nucleus for Weather Modification.” Journal of Weather Modification 21: 9–13.

[ppl70961-bib-0190] Whale, T. , M. Holden , T. Wilson , D. O'Sullivan , and B. Murray . 2018. “The Enhancement and Suppression of Immersion Mode Heterogeneous Ice‐Nucleation by Solutes.” Chemical Science 9: 4142–4151. 29780544 10.1039/c7sc05421a PMC5941198

[ppl70961-bib-0191] Willick, I. , L. V. Gusta , D. Fowler , and K. Tanino . 2019. “Ice Segregation in the Crown of Winter Cereals: Evidence for Extraorgan and Extratissue Freezing.” Plant, Cell & Environment 42: 701–7016. 10.1111/pce.13454 30291635

[ppl70961-bib-0192] Wipf, S. , and C. Rixen . 2010. “A Review of Snow Manipulation Experiments in Arctic and Alpine Tundra Ecosystems.” Polar Research 29: 95–109.

[ppl70961-bib-0193] Wisniewski, M. , C. Bassett , and L. V. Gusta . 2003. “An Overview of Cold Hardiness in Woody Plants: Seeing the Forest Through the Trees.” HortScience 38: 952–959.

[ppl70961-bib-0194] Wisniewski, M. , M. Fuller , D. Glenn , L. Gusta , J. Duman , and M. Griffith . 2002. “Extrinsic Ice Nucleation in Plants.” In Plant Cold Hardiness, 211–221. Springer US.

[ppl70961-bib-0195] Wisniewski, M. , L. V. Gusta , M. Fuller , and D. Karlson . 2009. Ice Nucleation, Propagation and Deep Supercooling: The Lost Tribes of Freezing Studies, 1–11. Plant Cold Hardiness.

[ppl70961-bib-0196] Wisniewski, M. , S. Lindow , and E. Ashworth . 1997. “Observations of Ice Nucleation and Propagation in Plants Using Infrared Video Thermography.” Plant Physiology 113: 327–334. 12223611 10.1104/pp.113.2.327 PMC158146

[ppl70961-bib-0197] Wisniewski, M. , G. Neuner , and L. V. Gusta . 2015. “The Use of High‐Resolution Infrared Thermography (HRIT) for the Study of Ice Nucleation and Ice Propagation in Plants.” Journal of Visualized Experiments 2015: 1–11. 10.3791/52703 PMC4542532 25992743

[ppl70961-bib-0198] Wisniewski, M. , I. Willick , J. Duman , D. Livingston , and S. Newton . 2020. “Plant Antifreeze Proteins.” In Antifreeze Proteins Volume, 1, Environment, Systematics and Evolution, 189–226. Springer International Publishing.

[ppl70961-bib-0199] Worthy, S. , A. Kumar , Y. Xi , et al. 2021. “The Effect of (NH_4_)_2_SO_4_on the Freezing Properties of Non‐Mineral Dust Ice‐Nucleating Substances of Atmospheric Relevance.” Atmospheric Chemistry and Physics 21: 14631–14648.

[ppl70961-bib-0200] Yamada, T. , K. Kuroda , Y. Jitsuyama , D. Takezawa , K. Arakawa , and S. Fujikawa . 2002. “Roles of the Plasma Membrane and the Cell Wall in the Responses of Plant Cells to Freezing.” Planta 215: 770–778. 12244442 10.1007/s00425-002-0814-5

[ppl70961-bib-0201] Yoshida, S. 1986. “Reverse Changes in Plasma Membrane Properties Upon Deacclimation of Mulberry Trees (Morus Bombysis Koidz.).” Plant and Cell Physiology 27: 83–89.

[ppl70961-bib-0202] Zámećník, J. , J. Bieblová , and M. Grospietsch . 1994. “Safety Zone as a Barrier to Root‐Shoot Ice Propagation.” Plant and Soil 167: 149–155.

[ppl70961-bib-0203] Zhang, Y. , S. Bucci , N. Arias , et al. 2016. “Freezing Resistance in Patagonian Woody Shrubs: The Role of Cell Wall Elasticity and Stem Vessel Size.” Tree Physiology 36: 1007–1018. 27217529 10.1093/treephys/tpw036

[ppl70961-bib-0204] Zobrist, B. , C. Marcolli , T. Peter , and T. Koop . 2008. “Heterogeneous Ice Nucleation in Aqueous Solutions: The Role of Water Activity.” Journal of Physical Chemistry A 112: 3965–3975. 18363389 10.1021/jp7112208

[ppl70961-bib-0205] Zweifel, R. , and R. Häsler . 2000. “Frost‐Induced Reversible Shrinkage of Bark of Mature Subalpine Conifers.” Agricultural and Forest Meteorology 102: 213–222.

